# Supplementary material for: De novo determination of mosquitocidal Cry11Aa and Cry11Ba structures from naturally-occurring nanocrystals
Source: Nat Commun. 2022 Jul 28;13:4376. doi: 10.1038/s41467-022-31746-x (PMC9334358; doi:10.1038/s41467-022-31746-x)
Supplement: Supplementary file 1 — Supplementary Information [file 41467_2022_31746_MOESM1_ESM.pdf]

Supplementary Information for:

De novo determination of mosquitocidal Cry11Aa and Cry11Ba structures from naturally-occurring nanocrystals

by

Guillaume Tetreau, Michael R. Sawaya, Elke De Zitter, Elena A. Andreeva, Anne-Sophie Banneville, Natalie A. Schibrowsky, Nicolas Coquelle, Aaron S. Brewster, Marie Luise Grünbein, Gabriela Nass Kovacs, Mark S. Hunter, Marco Kloos, Raymond G. Sierra, Giorgio Schiro, Pei Qiao, Myriam Stricker, Dennis Bideshi, Iris D. Young, Ninon Zala, Sylvain Engilberge, Alexander Gorel, Luca Signor, Jean-Marie Teulon, Mario Hilpert, Lutz Foucar, Johan Bielecki, Richard Bean, Raphael de Wijn, Tokushi Sato, Henry Kirkwood, Romain Letrun, Alexander Batyuk, Irina Snigireva, Daphna Fenel, Robin Schubert, Ethan J. Canfield, Mario M. Alba, Frédéric Laporte, Laurence Després, Maria Bacia, Amandine Roux, Christian Chapelle, François Riobé, Olivier Maury, Wai Li Ling, Sébastien Boutet, Adrian Mancuso, Irina Gutsche, Eric Girard, Thomas R. M. Barends, Jean-Luc Pellequer, Hyun-Woo Park, Arthur D. Laganowsky, Jose Rodriguez, Manfred Burghammer, Robert L. Shoeman, R. Bruce Doak, Martin Weik, Nicholas K. Sauter, Brian Federici, Duilio Cascio, Ilme Schlichting, Jacques-Philippe Colletier.

This PDF file contains:

- Supplementary Figures S1-S19
- Supplementary Tables S1-S4

```
                 10         20         30         40         50         60         70         80         90        100        110        120
                  |          |          |          |          |          |          |          |          |          |          |          |
Cry11Aa    MEDSSLDTLSIVNETDFPLYNNYTEPTIAPALIAVAPIAQYLATAIGKWAAKAAFSKVLSLIFPGSQPATMEKVRTEVETLINQKLSQDRVNILNAEYRGIIEVSDVFDAYIKQPGFTPA
Cry11Ba    MQNNNFNTTEINNMINFPMYNGRLEPSLAPALIAVAPIAKYLATALAKWAVKQGFAKLKSEIFPGNTPATMDKVRIEVQTLLDQRLQDDRVKILEGEYKGIIDVSKVFTDYVNQSKFETG
           *::..::* .* *  :**:**.  **::***********:*****:.***.* .*:*: * ****. ****:*** **:**::*:*.:***:**:.**:***:**.**  *::*. * ..

                130        140        150        160        170        180        190        200        210        220        230        240
                  |          |          |          |          |          |          |          |          |          |          |          |
Cry11Aa    TAKGYFLNLSGAIIQRLPQFEVQTYEGVSIALFTQMCTLHLTLLKDGILAGSAWGFTQADVDSFIKLFNQKVLDYRTRLMRMYTEEFGRLCKVSLKDGLTFRNMCNLYVFPFAEAWSLMR
Cry11Ba    TANRLFFDTSNQLISRLPQFEIAGYEGVSISLFTQMCTFHLGLLKDGILAGSDWGFAPADKDALICQFNRFVNEYNTRLMVLYSKEFGRLLAKNLNEALNFRNMCSLYVFPFSEAWSLLR
           **:  *:: *. :*.******:  ******:*******:** ********** ***: ** *::*  **: * :*.**** :*::*****    .*::.*.*****.******:*****:*

                250        260        270        280        290        300        310        320        330        340        350        360
                  |          |          |          |          |          |          |          |          |          |          |          |
Cry11Aa    YEGLKLQSSLSLWDYVGVSIP-VNYNEWGGLVYKLLMGEVNQRLTTVKFNYSFTNEPADIPARENIRGVHPIYDPSSGLTGWIGNGRTNNFNFADNNGNEIMEVRTQTFYQNP--NNEPI
Cry11Ba    YEGTKLENTLSLWNFVGESINNISPNDWKGALYKLLMGAPNQRLNNVKFNYSYFSDTQATIHRENIHGVLPTYNGGPTITGWIGNGRFSGLSFPCSNELEITKIKQEITYNDKGGNFNSI
           *** **:.:****::** **  :. *:* * :******  ****..******: .:.     ****:** * *: .. :******** ..:.*. .*  ** ::: :  *::   * :.*

                370        380        390        400        410        420        430        440        450        460        470        480
                  |          |          |          |          |          |          |          |          |          |          |          |
Cry11Aa    APRDIINQILTAPAP--ADLFFKNADINVKFTQWFQSTLY-GWNIKLGTQTVLSSRTG-TIPPNYLAYDGYYIRAISACPRGVSLAYNHDLTTLTYNRIEYDSPTTENIIVGFAPDNTKD
Cry11Ba    VPAATRNEILTATVPTSADPFFKTADINWKY---FSPGLYSGWNIKFDDTVTLKSRVPSIIPSNILKYDDYYIRAVSACPKGVSLAYNHDFLTLTYNKLEYDAPTTQNIIVGFSPDNTKS
           .*    *:****..*  ** ***.**** *:   *.. ** *****:.  ..*.**.  **.* * **.*****:****:*********: *****::***:***:******:*****.

                490        500        510        520        530        540        550        560        570        580        590        600
                  |          |          |          |          |          |          |          |          |          |          |          |
Cry11Aa    FYSKKSHYLSETNDSYVIPALQFAEVSDRSFLEDTPDQATDGSIKFARTFISNEAKYSIRLNTGFNTATRYKLIIRVRVPYRLPAGIRVQSQNSGNNRMLGSFTANANPEWVDFVTDAFT
Cry11Ba    FYRSNSHYLSTTDDAYVIPALQFSTVSDRSFLEDTPDQATDGSIKFTDTVLGNEAKYSIRLNTGFNTATRYRLIIRFKAPARLAAGIRVRSQNSGNNKLLGGIPVEGNSGWIDYITDSFT
           **  .:***** *:*:********: ********************: *.:.******************:****.:.* **.*****:*******::**.:..:.*. *:*::**:**

                610        620        630        640        650        660        670        680        690        700        710        720    727
                  |          |          |          |          |          |          |          |          |          |          |          |      |
Cry11Aa    FNDLGITTSSTNALFSISSDSLNSGEEWYLSQLFLVKESAFTTQI--NPLLK------------------------------------------------------------------------
Cry11Ba    FDDLGITTSSTNAFFSIDSDGVNASQQWYLSKLILVKESSFTTQIPLKPYVIVRCPDTFFVSNNSSSTYEQGYNNNYNQNSSSMYDQGYNNSYNPNSGCTCNQDYNNSYNQNSGCTCNQGYNNNYPK
           *:***********:***.**.:*:.::****:*:*****:*****  :* :
```

Supplementary Fig. 1. Cry11Aa and Cry11Ba exhibit high similarity but limited sequence identity. Cry11Aa and Cry11Ba were aligned using strap [97]. This shows that 54.0% (392 residues) of Cry11Aa and Cry11Ba residues are identical. Domain I is shown in blue; domain II is shown in orange except for the $\alpha_h\beta_h$-handle and β_{pin} which are shown in purple and red, respectively; domain III is shown in pink.

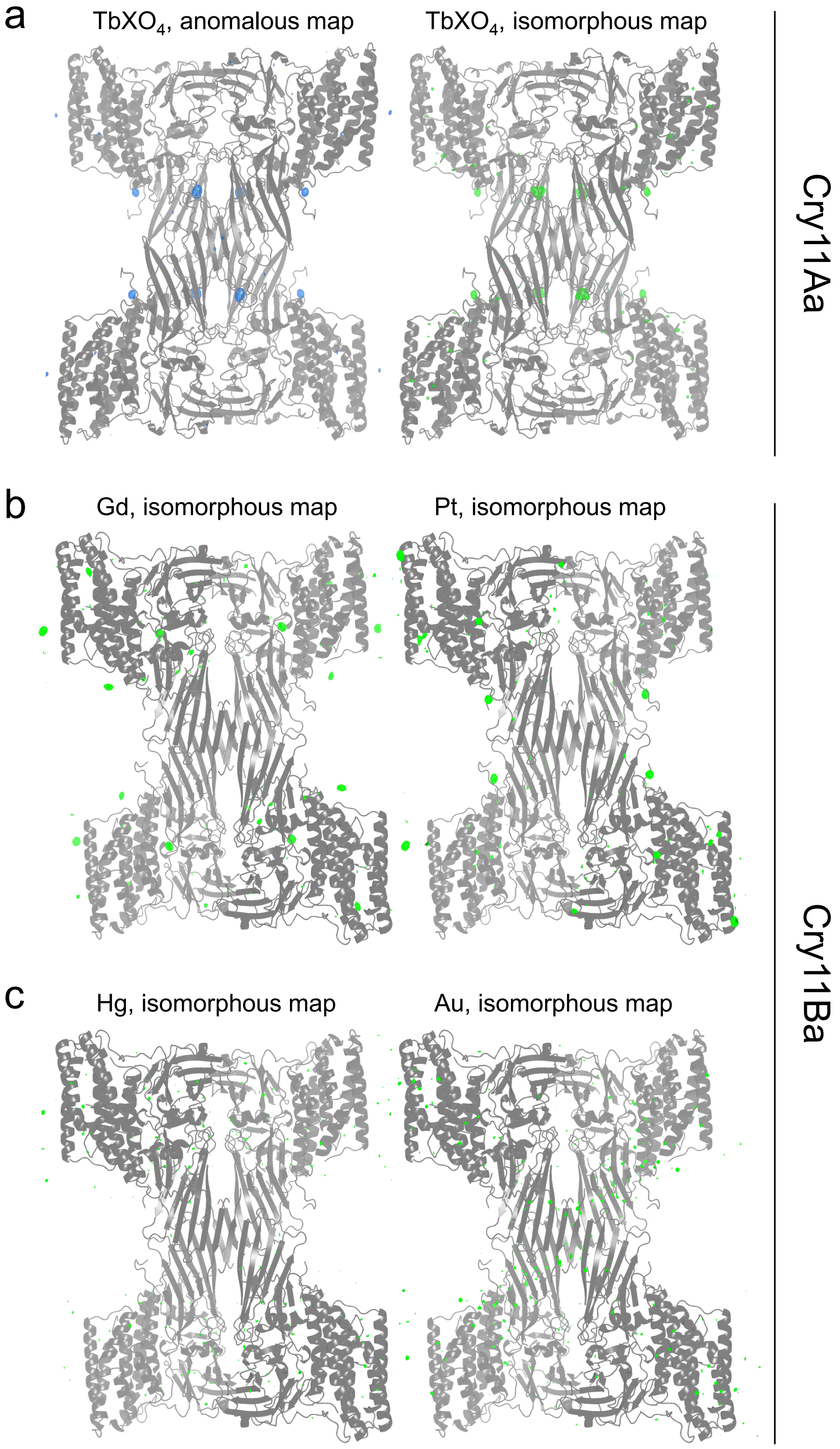

Fig. 2. Heavy atom locations after soaking of the Cry11 crystals. a, Location of Tb-Xo4 in Cry11Aa is clearly indicated the anomalous (left; highest peak at 33.1 σ) and isomorphous difference maps (right; highest peak at 24.5 σ), both contoured at ± 4 σ. **b-c**, Native Cry11Ba structure revealed a posteriori that soaking of Cry11Ba with salts of gadolinium and platinum (**b**) led to their successful binding to the crystalline Cry11Ba, as revealed by isomorphous peaks > 9.3 and 7.5 σ, respectively. However, no anomalous signal was detected (highest peaks at 5.5 and 5.0 σ, respectively). Soaking of Cry11Ba crystals with salts mercury or gold was fully infructuous, with significant peaks visible neither in the isomorphous (highest peaks at 5.3 and 5.5 σ, respectively) nor the anomalous (highest peaks at 4.9 and 5.5 σ, respectively) maps. In (**b**) and (**c**), the isomorphous difference maps are contoured at ± 4 σ.

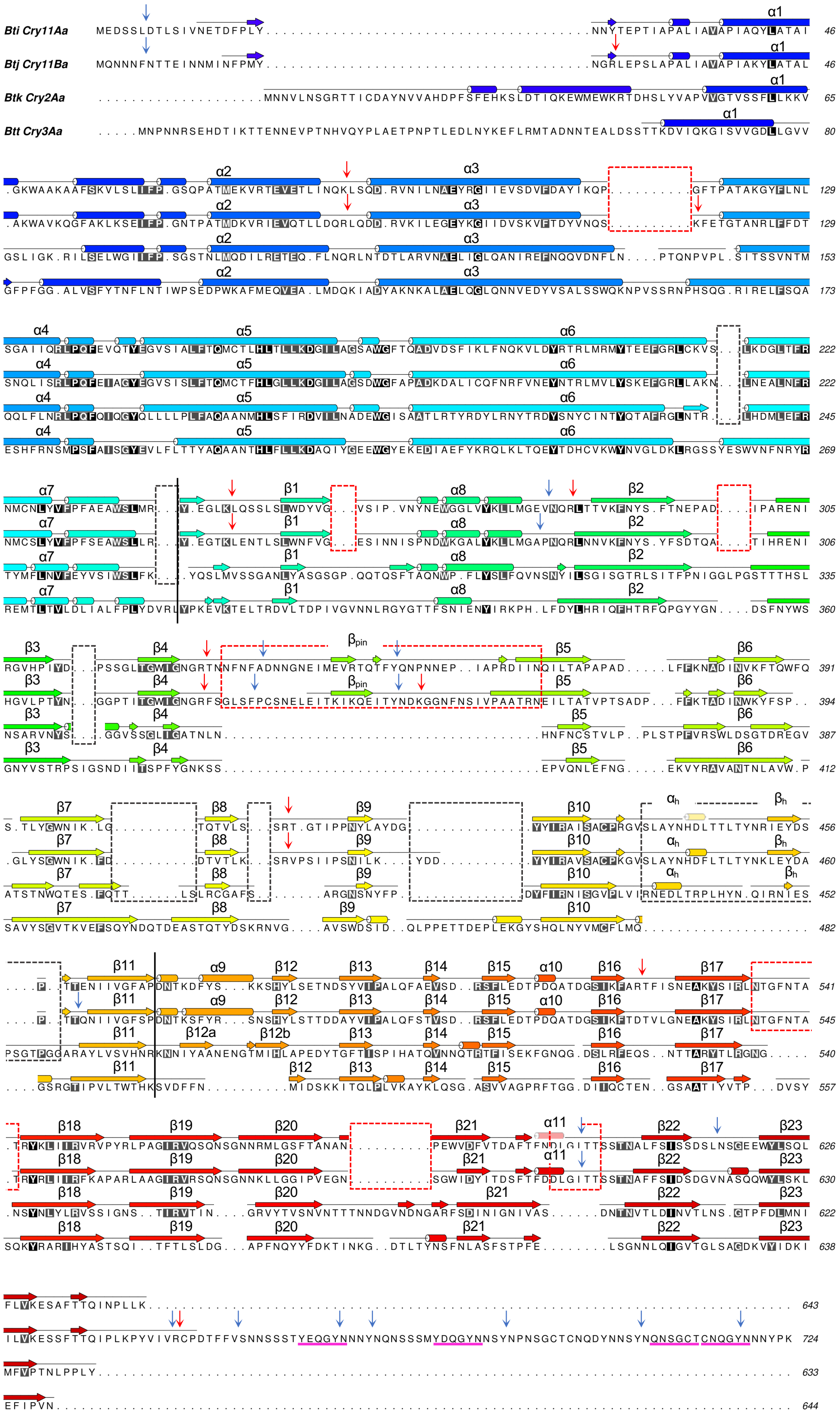

Supplementary Fig. 3. Secondary structure assignment of Cry11Aa, Cry11Ba, Cry2Aa and Cry3Aa. Secondary structures were assigned using DSSP[103] and colored according to sequence (from blue to red). α-helices and β-strand are shown by rods and arrows, respectively. Vertical black lines show the domain borders. Remarkable regions of difference between the Cry11 toxins and all other Cry toxins are indicated by red dashed boxes, whereas black boxes indicate differences between Cry11 and Cry2Aa toxin as compared to other toxins. Vertical red and blue arrows indicate trypsin and proteinase K digestion sites, respectively. Regions predicted to form short adhesive motifs of the Low Complexity, Amyloid-like Reversible Kinked Segments (LARKS) type are underlined in magenta.

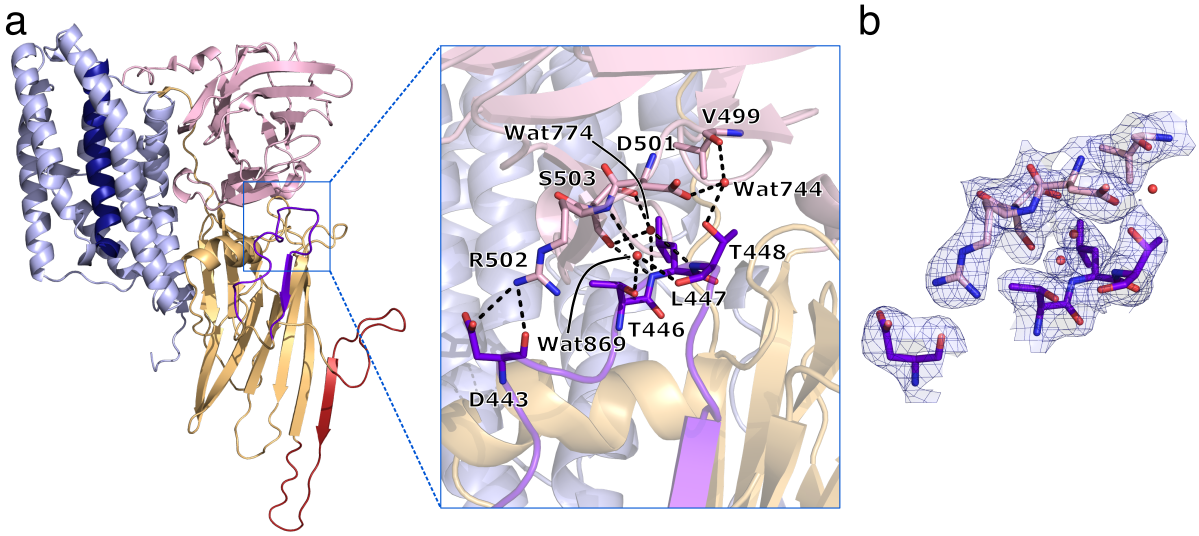

Supplementary Fig. 4. Interactions by the $\alpha_h\beta_h$-handle in Cry11Aa. Color code as in Fig. 2. **a**, Dashes indicate hydrogen bonds (up to 3.2 Å) and the salt bridge between D443 and R502. **b**, $2F_{obs}$-DF_{calc} electron density map for the residues and water molecules shown in (**a**), contoured at 1 σ. Water molecule 744 is defined in the map with a contour level of 0.9 σ but not at a level of 1 σ.

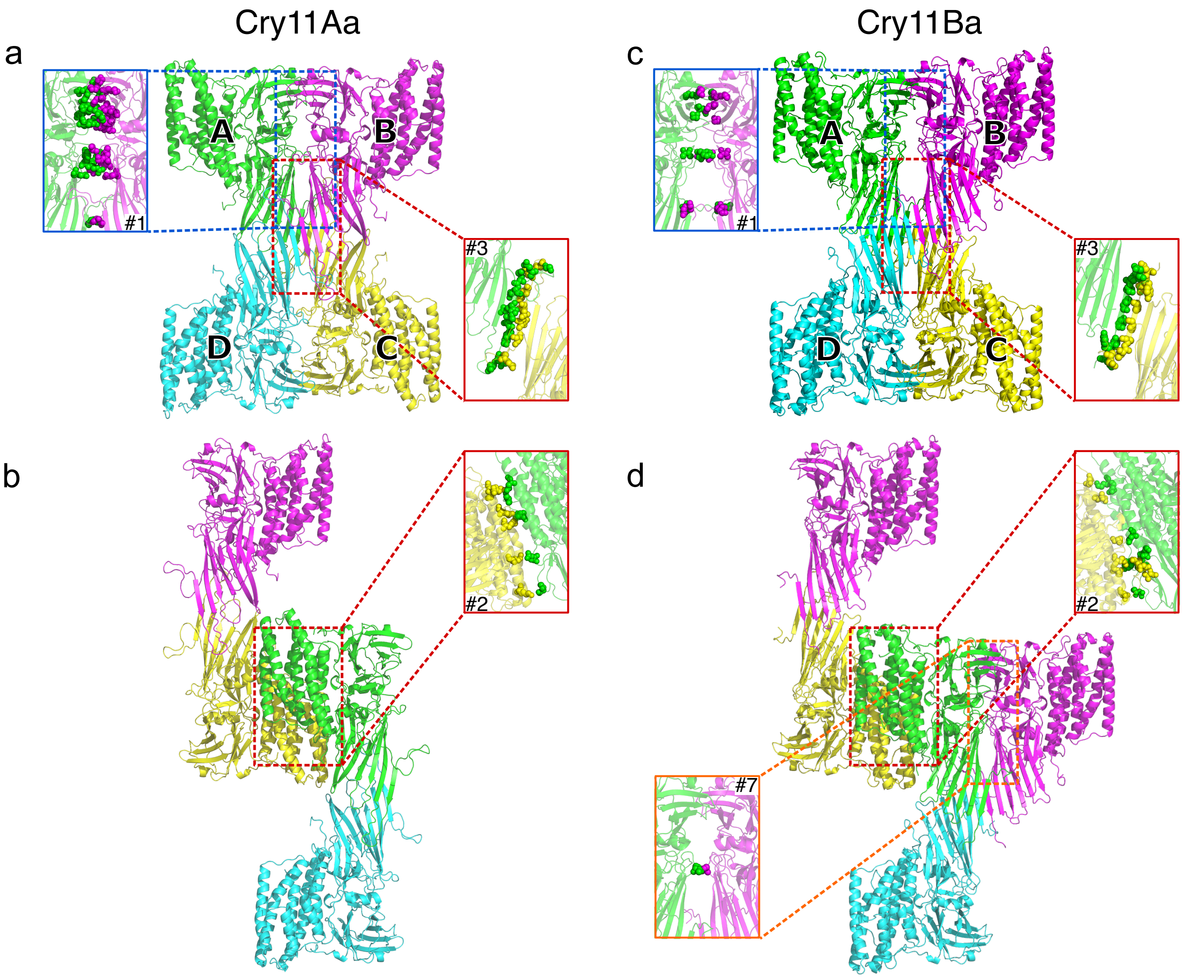

Supplementary Fig. 5. Hydrogen bonds and salt bridges between monomer in Cry11Aa and Cry11Ba crystals. Colour code as in Fig. 3. **a**, Cry11Aa tetramer with zoom on each of the interfaces identified by PISA that contain hydrogen bonds or salt bridges identified by PISA (interface #1 and #3), with the residues involved in these interactions depicted as spheres. **b**, In the Cry11Aa crystal assembly between neighbouring tetramers only interface #2 contains hydrogen and salt bridges. These are visualized as in (**a**). **c**, Cry11Ba tetramer with zoom on the interfaces that contain salt bridges and hydrogen bonds as in (**a**). **d**, Cry11Ba crystal assembly between neighbouring tetramers, visualized as in (**b**).

a

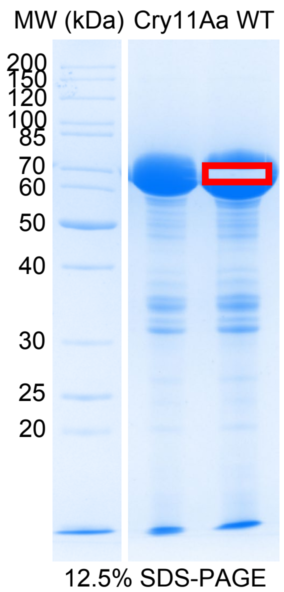

Database: SwissProt
Protein View: C11AA_BACTI (P21256)
Score (Mascot): 191
Protein coverage: 45% (matched peptides in bold red)

1 MEDSSLDTLS IVNETDFPLY NNYTEPTIAP ALIAVAPIAQ YLATAIGKWA
51 AKAAFSK**VLS LIFPGSQPAT MEK**VRTEVET LINQKLSQDR **VNILNAEYRG**
101 **IIEVSDVFDA YIK**QPGFTPA TAK**GYFLNLS GAIIQR**LPQF EVQTYEGVSI
151 ALFTQMCTLH LTLLK**DGILA GSAWGFTQAD VDSFIK**LFNQ KVLDYRTRLM
201 R**MYTEEFGR**L CKVSLK**DGLT FRNMCNLYVF PFAEAWSLMR** YEGLKLQSSL
251 SLWDYVGVSI PVNYNEWGGL VYK**LLMGEVN QR**LTTVK**FNY SFTNEPADIP**
301 **AR**ENIRGVHP IYDPSSGLTG WIGNGRTNNF NFADNNGNEI MEVR**TQTFYQ**
351 **NPNNEPIAPR DIINQILTAP APADLFFK**NA DINVK**FTQWF QSTLYGWNIK**
401 LGTQTVLSSR **TGTIPPNYLA YDGYYIR**AIS ACPR**GVSLAY NHDLTTLTYN**
451 **RIEYDSPTTE NIIVGFAPDN TK**DFYSK**KSH YLSETNDSYV IPALQFAEVS**
501 **DRSFLEDTPD QATDGSIK**FA RTFISNEAKY SIR**LNTGFNT ATR**YKLIIRV
551 RVPYR**LPAGI R**VQSQNSGNN RMLGSFTANA NPEWVDFVTD AFTFNDLGIT
601 TSSTNALFSI SSDSLNSGEE WYLSQLFLVK ESAFTTQINP LLK

b

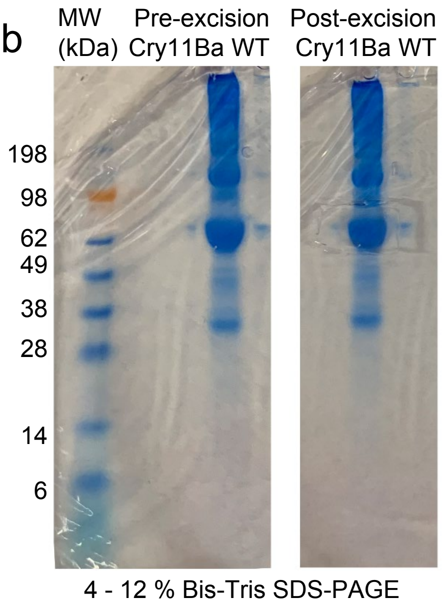

Database: SwissProt
Protein View: C11BA_BACTJ (Q45730)
Score (Mascot): 916.64
Protein coverage: 74% (matched peptides in bold red)

1 MQNNNFNTTE INNMINFPMY **NGRLEPSLAP ALIAVAPIAK YLATALAKWA**
51 **VKQGFAKLKS EIFPGNTPAT MDKVRIEVQT LLDQRLQDDR VKILEGEYKG**
101 **IIDVSKVFTD YVNQSKFETG TANRLFFDTS NQLISR**LPQF EIAGYEGVSI
151 SLFTQMCTFH **LGLLKDGILA GSDWGFAPAD KDALICQFNR FVNEYNTRLM**
201 **VLYSKEFGRL LAKNLNEALN FRNMCSLYVF PFSEAWSLLR YEGTKLENTL**
251 **SLWNFVGESI NNISPNDWKG ALYKLLMGAP NQRLNNVKFN YSYFSDTQAT**
301 **IHRENIHGVL PTYNGGPTIT GWIGNGRFSG LSFPCSNELE ITKIKQEITY**
351 **NDKGGNFNSI VPAATRNEIL TATVPTSADP FFKTADINWK YFSPGLYSGW**
401 **NIKFDDTVTL KSRVPSIIPS NILKYDDYYI RAVSACPKGV SLAYNHDFLT**
451 **LTYNKLEYDA PTTQNIIVGF SPDNTK**SFYR **SNSHYLSTTD DAYVIPALQF**
501 **STVSDRSFLE DTPDQATDGS IKFTDTVLGN EAKYSIRLNT GFNTATRYRL**
551 **IIRFKAPARL AAGIRVRSQN SGNNK**LLGGI PVEGNSGWID YITDSFTFDD
601 LGITTSSTNA FFSIDSDGVN **ASQQWYLSKL ILVKESSFTT QIPLKPYVIV**
651 **R**CPDTFFVSN NSSSTYEQGY NNNYNQNSSS MYDQGYNNSY NPNSGCTCNQ
701 DYNNSYNQNS GCTCNQGYNN NYPK

Supplementary Fig. 6. SDS PAGE and mass spectrometry confirm that Cry11Aa and Cry11Ba crystals are solely composed of these proteins. a, The most abundant band present at ~70 kDa from the proteomic profile of Cry11Aa purified crystal suspension was cut and digested with protease before being analyzed in MALDI. Analysis using Mascot Software allowed matching the majority of peptides identified, covering 45 of the Cry11Aa sequence (indicated in red). **b,** The most abundant band present at ~ 80 kDa from the SDS PAGE gel of the was digested with trypsin before being analyzed by LC-MS/MS. Analysis using Mascot Software allowed matching the majority of peptides identified, covering 74% of the Cry11Ba sequence (indicated in red). The MALDI experiment on Cry11Aa was performed once, and that on Cry11Ba was performed twice. Proteomic profiling by SDS-PAGE was performed more than ten times on both samples.

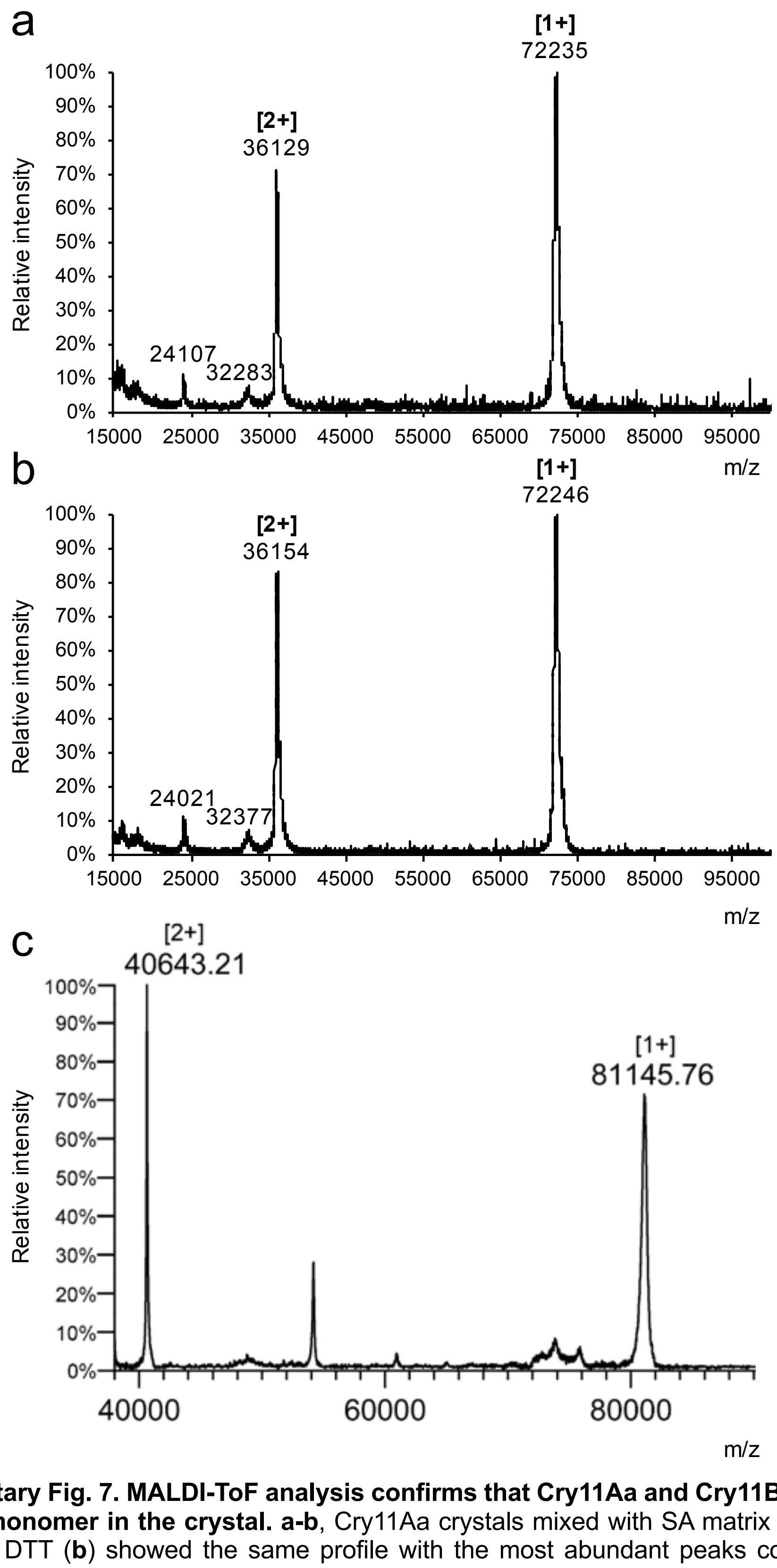

Supplementary Fig. 7. MALDI-ToF analysis confirms that Cry11Aa and Cry11Ba are present as a full-size monomer in the crystal. a-b, Cry11Aa crystals mixed with SA matrix in absence (**a**) or presence of DTT (**b**) showed the same profile with the most abundant peaks corresponding to a monomer of 72,235–72,246 kDa (expected mass: 72,349 kDa) monocharged or bicharged. **c**, Cry11Ba crystals mixed with DHAP showed the presence of full-length monomer of 81,145 kDa in agreement with the predicted mass of 81,344 kDa.

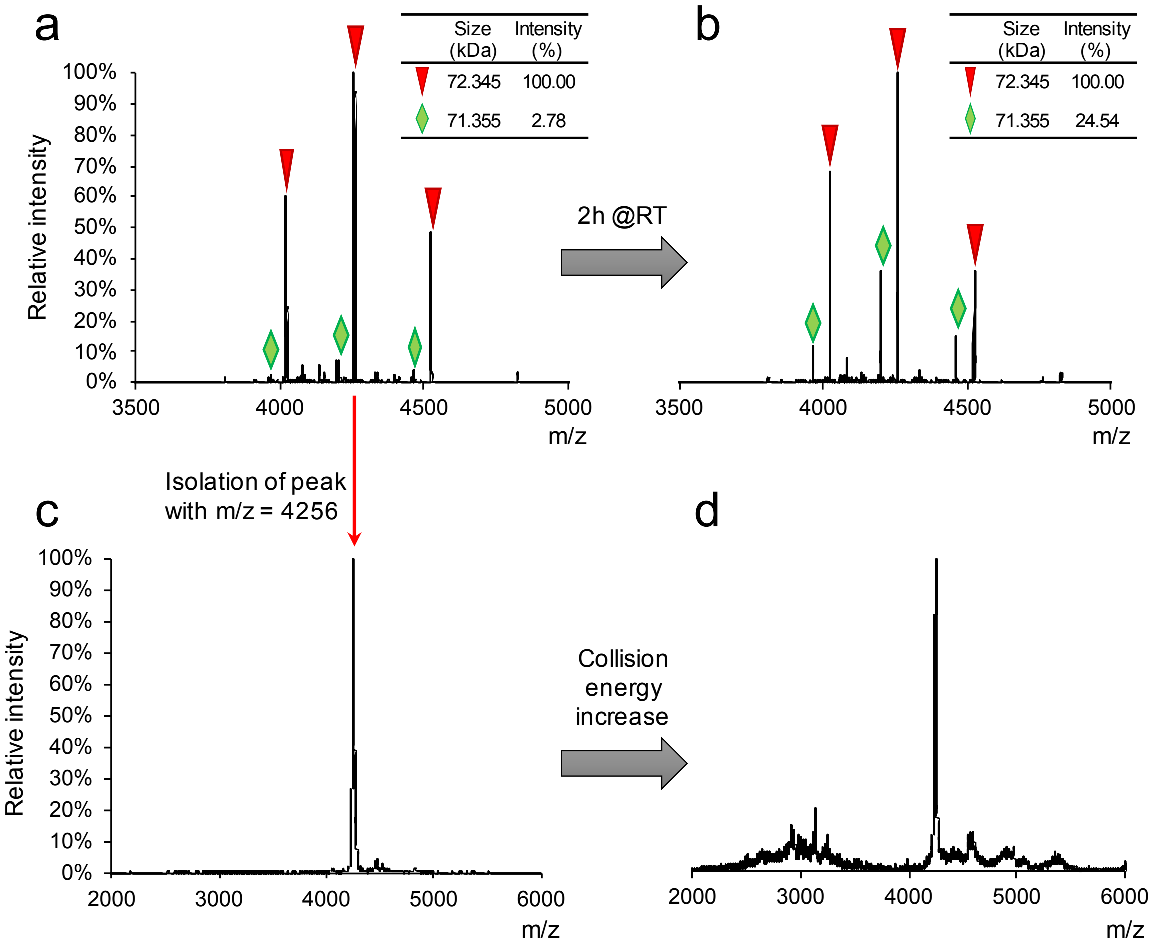

Supplementary Fig. 8. Native mass spectrometry confirms that Cry11Aa is solubilized as a full-size monomer. a, profile of soluble Cry11Aa in native MS shows three peaks corresponding to a full size toxin of 72.345 kDa (triangles, expected mass: 72.349 kDa) and three peaks for a ~1 kDa smaller form much less abundant (diamonds), likely resulting from a cleavage of the first 9 amino acids in N-terminal part of the toxin. **b**, incubation at room temperature leads to an increase in the smaller form, reaching one fourth of abundance of full size one, suggesting a targeted cleavage of few amino acids in the N- and/or C-terminal extremity. **c**, when isolating the most abundant peak (*i.e.,* at m/z = 4256, corresponding to Cry11Aa with a charge of 17) in the same condition as in panel a, the peak is sharp and well defined. **d**, increasing the collision energy leads to a fragmentation into species with different sizes. If the protein is an oligomer with multiple monomers through non-covalent bound, specific monomers would be easily separated and identified on the MS spectrum. This rather supports that Cry11Aa solubilizes as a full-sized monomer prone to degradation under certain conditions.

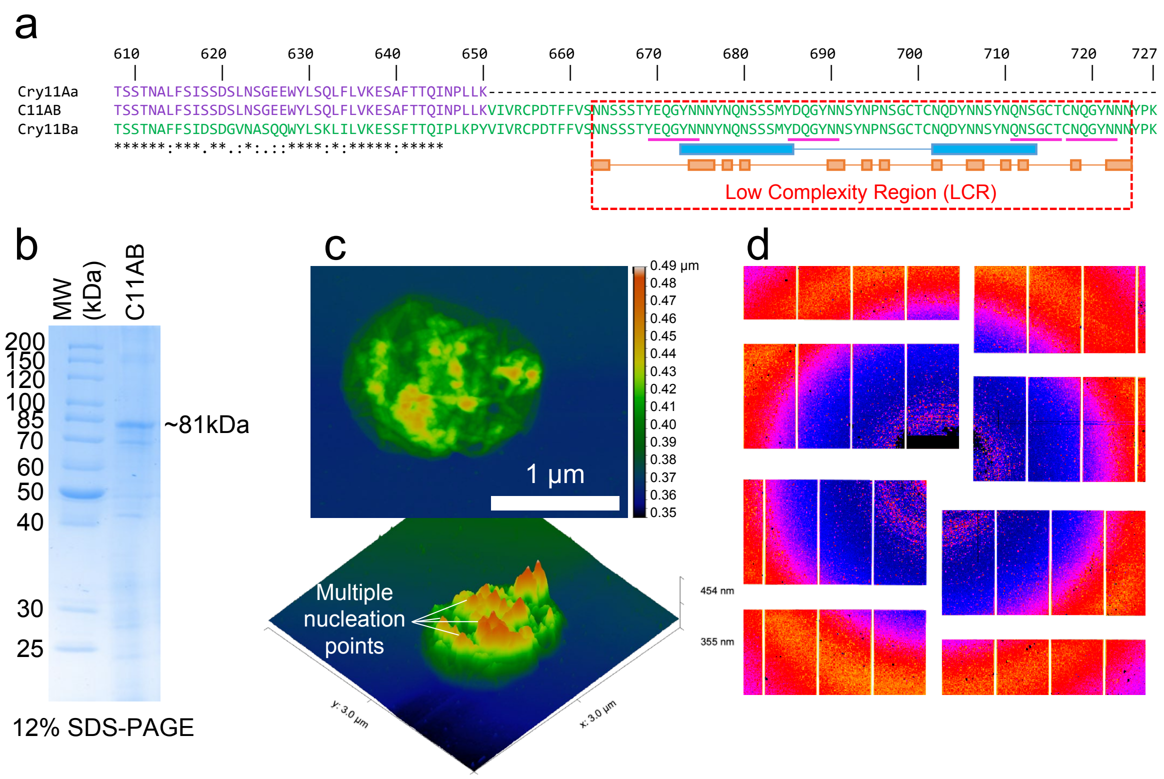

Supplementary Fig. 9. Addition of the Cry11Ba C-terminal Low Complexity Region (LCR) to Cry11Aa does not improve its crystallization. a, amino acid sequence alignment of Cry11Aa, Cry11Ba and Cry11Aa fused with the last 77 residues of Cry11Ba (named C11AB). Residues originating from Cry11Aa and Cry11Ba are indicated in purple and green, respectively, Blue and orange rectangles represent LCR domains identified by SEG and CAST programs, respectively. Segments predicted to form larks are underlined in magenta. **b**, C11AB crystals were purified using sucrose gradients and verified on 12% SDS-PAGE gels yielding a major band at a mean of 81.5 kDa, which matches the expected size for the fusion, although expressed at a lower level than Cry11Aa WT. **c**, purified inclusions analyzed by AFM revealed multiple nucleation points instead of a unique microcrystal. The experiment was performed once. **d**, maximum projection of the few diffraction patterns that were obtained using the C11AB inclusions at the EuXFEL indicating only limited diffraction.

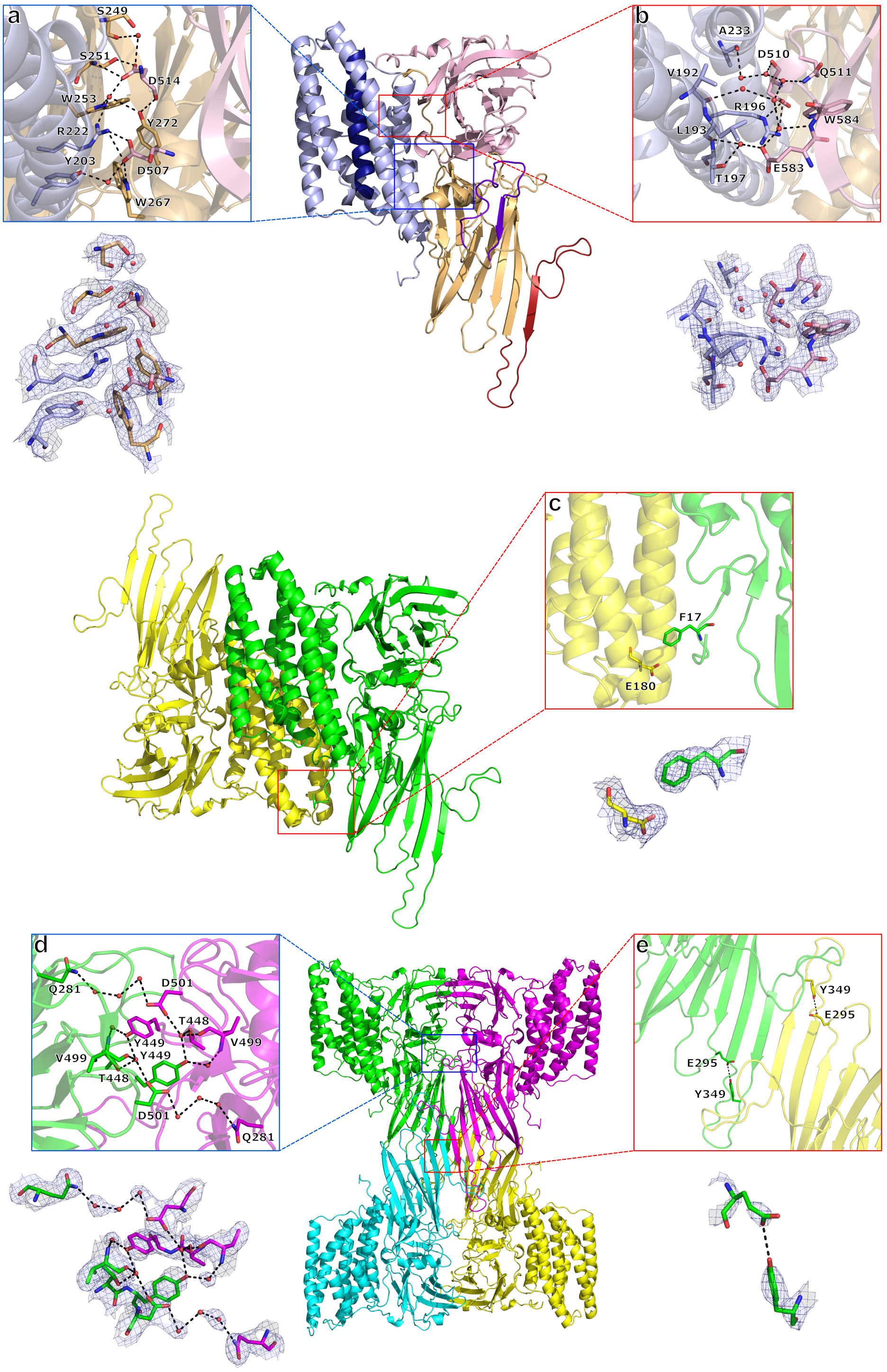

Supplementary Fig. 10. Cry11Aa mutation strategy. a, Hydrogen and salt bonding interaction network between domain I, II and III within a single monomer formed by Y272, D507 and D514. Color code as in Fig. 2. For clarity, a part of domain III is not shown in cartoon mode. Hydrogen bonds are shown up until a distance of 3.2 Å. The $2F_{obs}$-F_{calc} electron density map contouring the concerned residues and water molecules at 1 σ is shown at the bottom of each panel. **b**, Hydrogen and salt bonding interaction network between domain I and III within a single monomer involving E583. Color code as in Fig. 2. **c**, Location of F17 and E180 in interface #2 between two adjacent monomers. Monomers are color coded as in Fig. 3. **d**, Water-mediated hydrogen bonding network around Y449 in interface #1 between two adjacent monomers. For clarity, domain III of each of the monomer is omitted from the figure. Color code as in Fig. 3. **e**, Interaction between E295 and Y349 in the β_{pin} region which connects two adjacent monomers via interface #3. Color code as in Fig. 3.

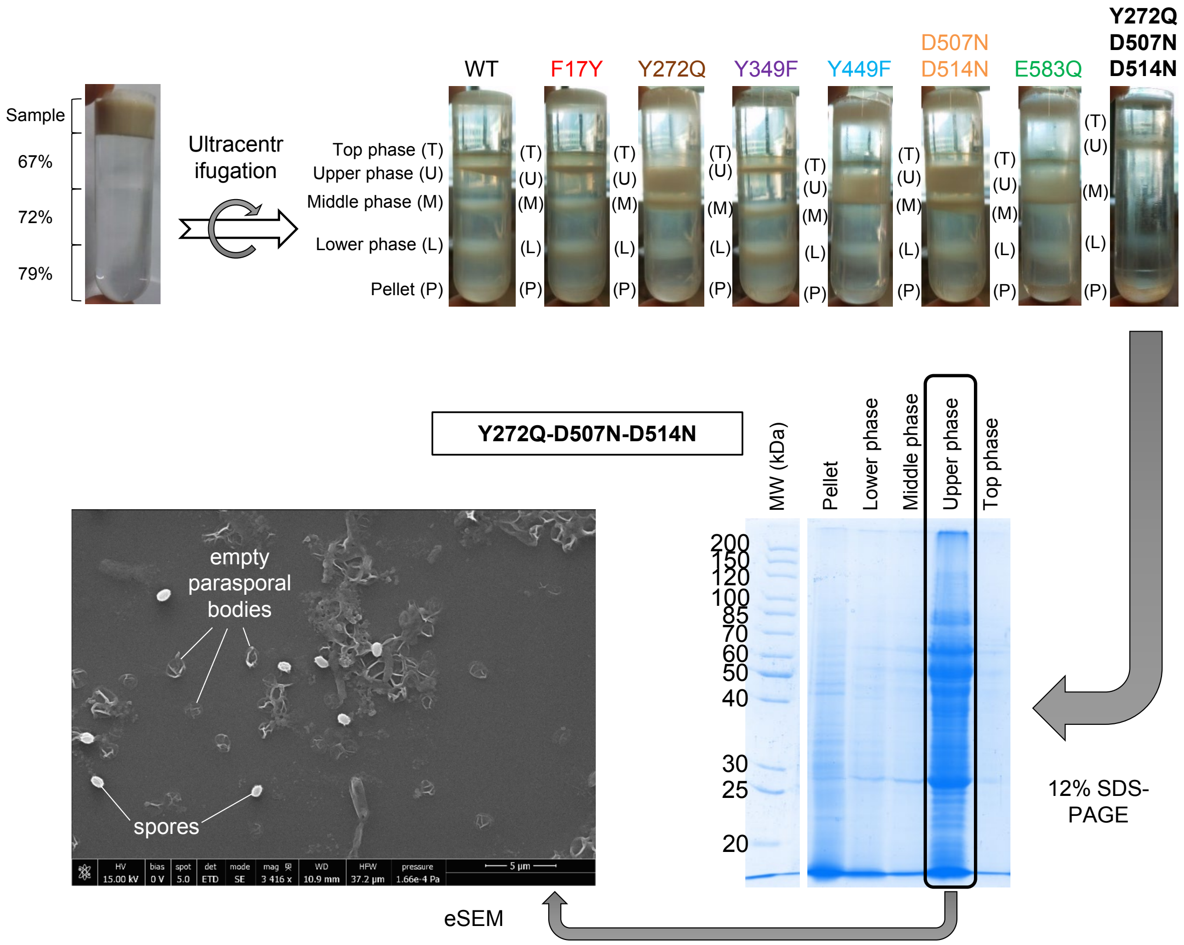

Supplementary Fig. 11. The triple mutation Y272Q-D507N-D514N of Cry11Aa affects crystal formation. All Cry11Aa mutants with one and two point-mutations (D507N-D514N) produced crystals that could be properly purified by sucrose gradient coupled with ultracentrifugation. In contrast, the triple mutant Y272Q-D507N-D514N showed a different purification profile. Most of proteins were contained in the upper phase of the sucrose gradient but no clear band at the ~70 kDa size could be seen. This is confirmed by environmental SEM (eSEM) experiments showing that this phase mostly contains empty parasporal bodies, suggesting that this mutation is deleterious for the proper production and/or crystallization of the toxin in the bacterium during its sporulation. The eSEM imaging of the Y272Q-D507N-D514N mutant was performed once.

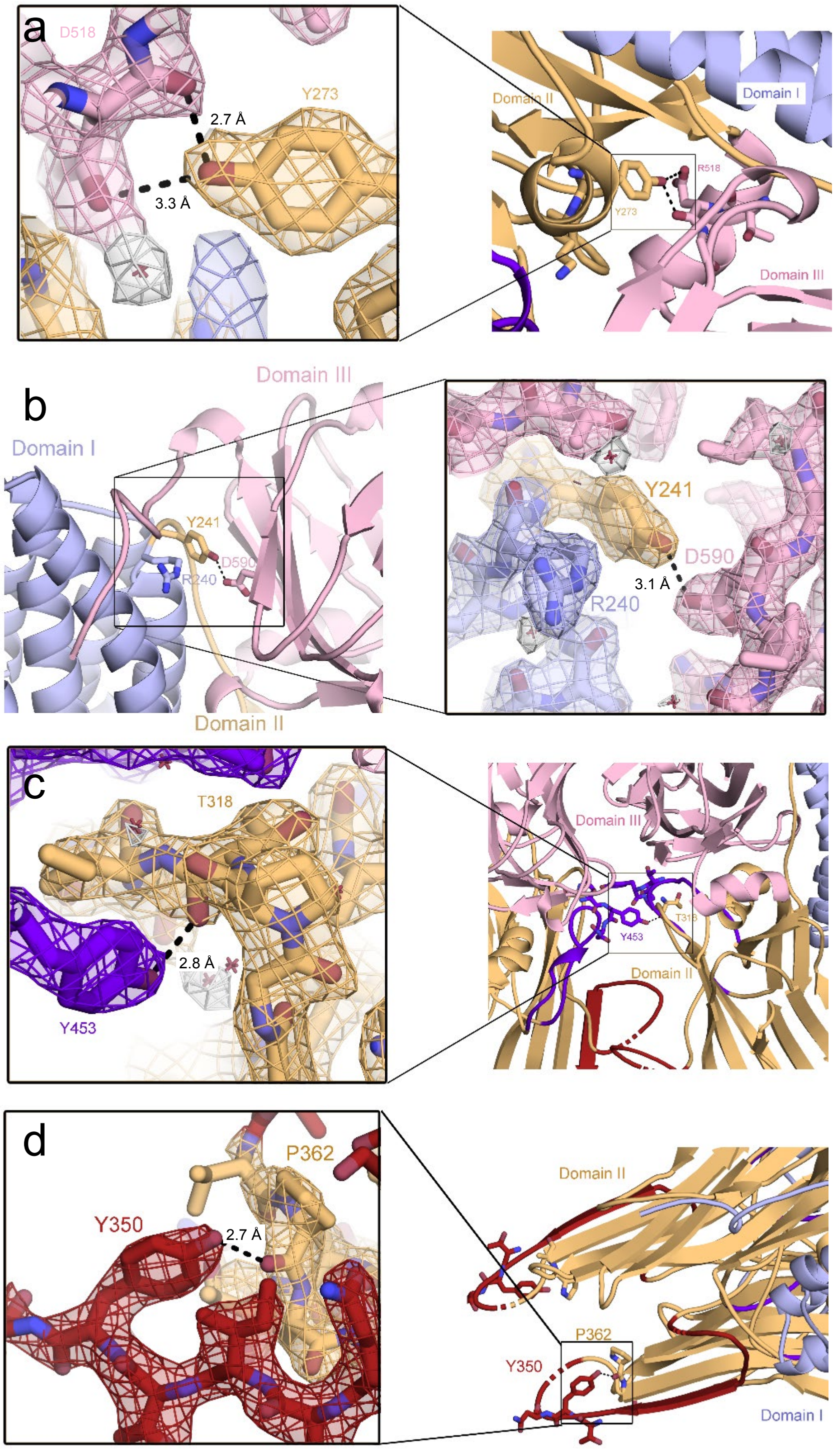

Supplementary Fig. 12. Cry11Ba structure with close-ups of electron density at selected mutation sites. Color code as in Fig. 2. **a**, The Y273F mutation was selected to disrupt the H-bonding of the Y273 to the R518 backbone carbonyl (~2.6 Å) and the intermittent H-bond of D517 side chain carbonyl (~3.3 Å) on the same chain between domain interfaces. **b**, The Y241F mutation causes the disruption of the H-bond (2.8 Å) between the Y241 hydroxyl and D590 carbonyl group, which is between two domain interfaces. **c**, The Y453F mutation caused a loss of the Y453's hydroxyl group H-bond to T318's carbonyl backbone of a different chain, which is located at an interface. **d**, Y350 is conserved in Cry11Ba where it H-bonds to P362(O), and the equivalent Y350F mutant solubilized at a lower pH.

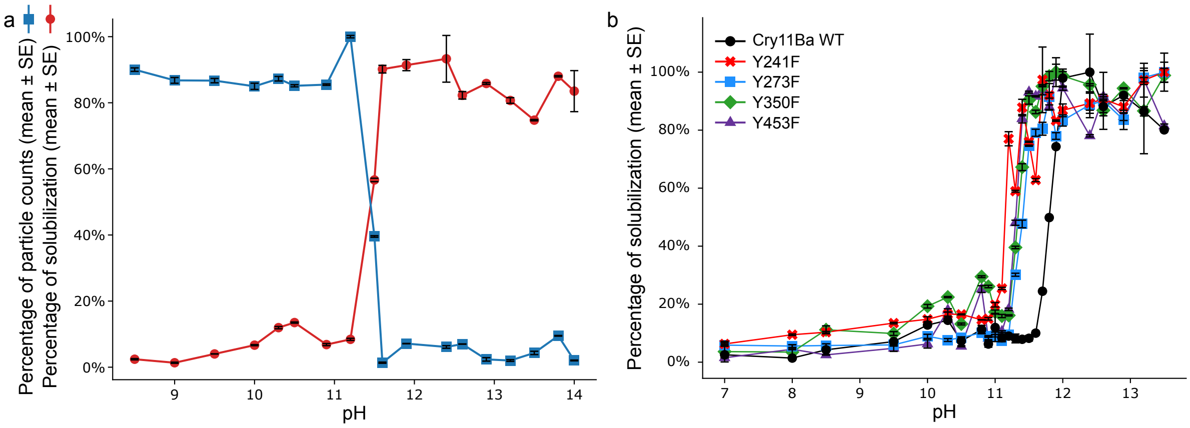

Supplementary Fig. 13. Cry11Ba and Cry11Ba mutants crystal solubilization and stabilization in function of pH. a, Solubility (red circles) and turbidity (blue squares) assay on Cry11Ba crystals indicate that 50 % of crystals solubilize at pH ~ 11.9 (n=3 independent measurements, data are presented as mean values +/- SEM). **b**, Solubility of Cry11Ba WT (black circles) and mutants (Y241F: red crosses, Y273F: blue squares, Y350F: green diamonds, Y453F: purple triangles) in function of pH show that the mutants solubilize at lower pH (~ 11.3) than the WT(n=3 independent measurements, data are presented as mean values +/- SEM).

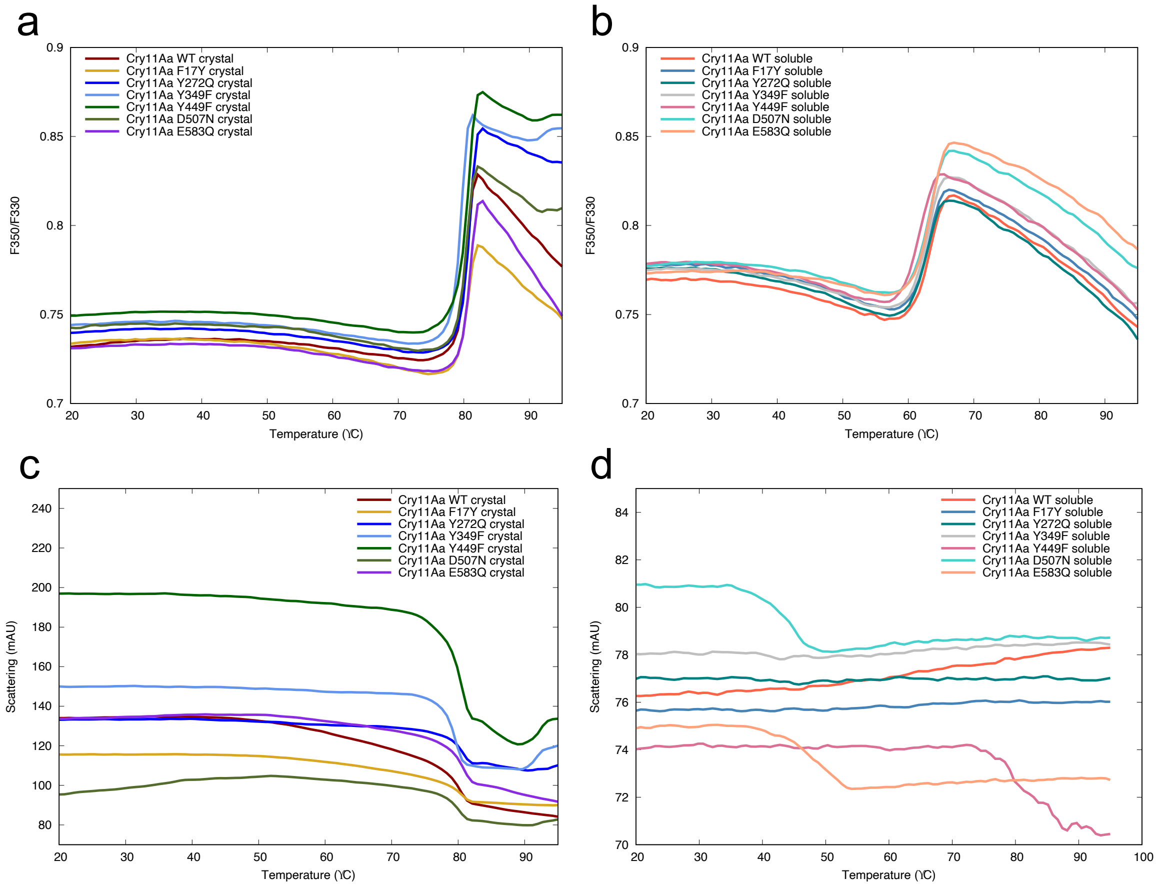

Supplementary Fig. 14. Toxin state (crystal *vs* soluble) but not point-mutations affected the thermal stability and aggregation propensity of Cry11Aa. a-b, Differential scanning fluorometry measurements indicate the thermal unfolding of crystals (**a**) and soluble (**b**) Cry11Aa WT and mutants in function of the temperature. **c-d**, Scattering measurement indicates aggregation propensity of crystals (**c**) and soluble toxins (**d**).

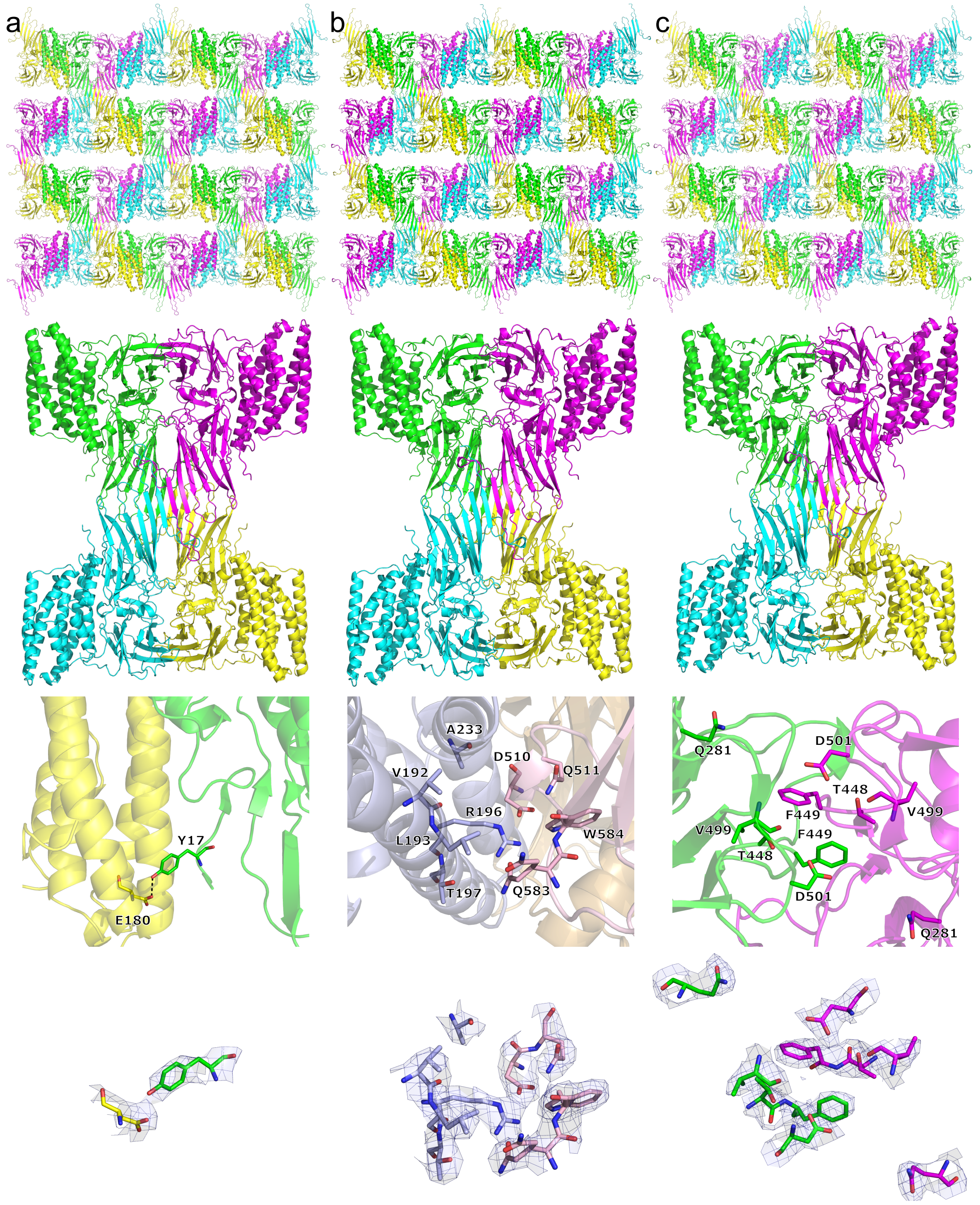

Supplementary Fig. 15. Cry11Aa mutant crystal structures indicating the crystal packing (top), tetramer (middle) and interaction region (bottom). Color code as in Fig. 2 and 3. **a**, Cry11Aa-F17Y; **b**, Cry11Aa-E583Q; **c**, Cry11Aa-Y449F. The dashes in (**a**) indicate the hydrogen bond made between E180(OE1) and Y17(OH) (2.4 Å). Due to the lower resolution as compared to Cry11Aa-WT, no water molecules are observed in the interaction region. Therefore, the specific interaction pattern is not shown in (**b**) and (**c**). The $2F_{obs}$-F_{calc} electron density map shown at the bottom of each panel is contoured at 1 σ.

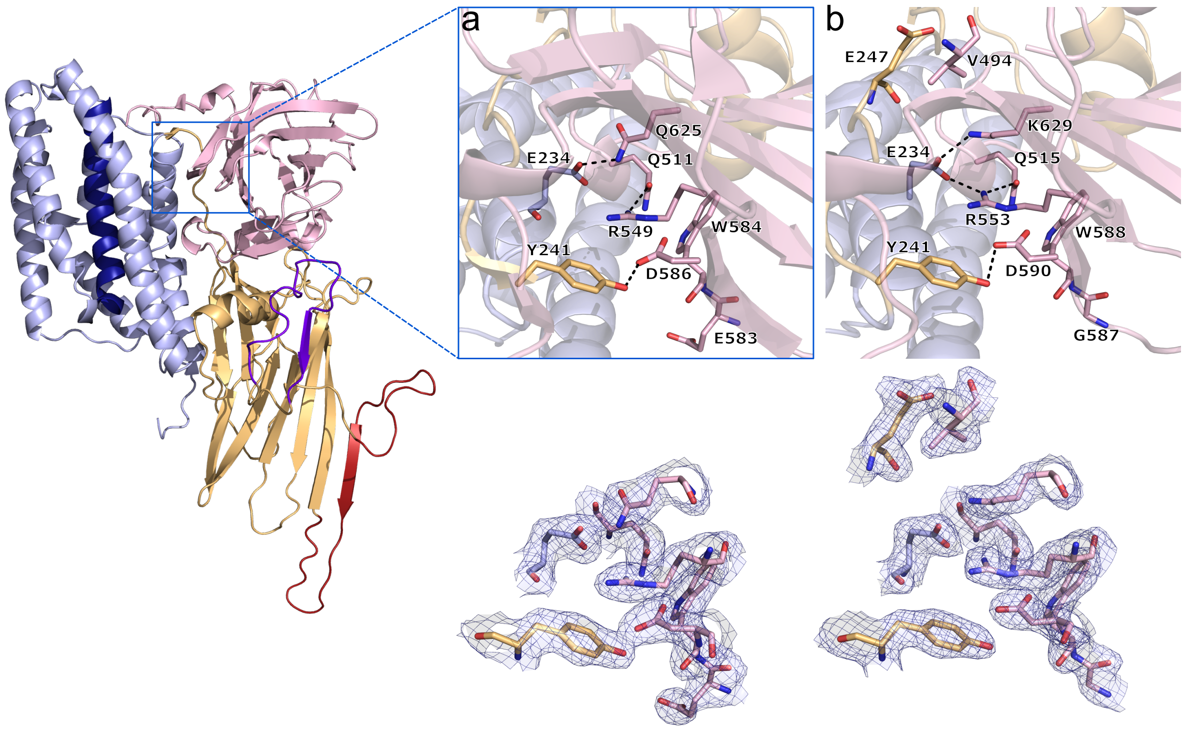

Supplementary Fig. 16. Potential mutation candidates to destabilize the domain I – domain III interface. Interactions between the concerned residues in chain A of Cry11Aa (**a**) and Cry11Ba (**b**), respectively. Color code as in Fig. 2. Dashes indicate hydrogen bonds or salt bridges (up to 3.2 Å). The $2F_{obs}$-F_{calc} map on the bottom of each panel is contoured at 1 σ.

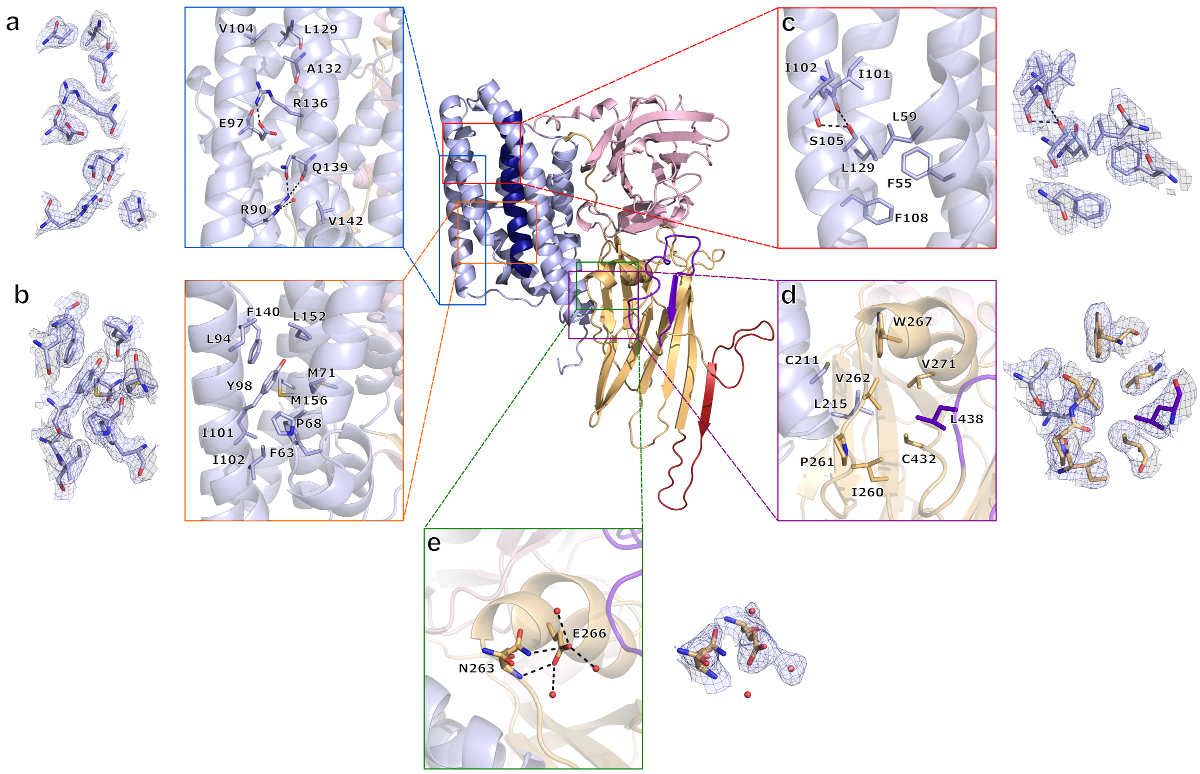

Supplementary Fig. 17. Structural interpretation of Cry11Aa mutations described in literature. Color code as in Fig. 2. Dashes indicate hydrogen bonds (up to 3.2 Å). The $2F_{obs}$-F_{calc} electron density maps on the left and right of each panel are contoured at 1 σ. **a**, Position of and interactions formed by residues in Domain I which can explain the suppression of toxicity of the V104E mutation, and reduced toxicity by the E97A, R90E and V142E mutations. **b**, Hydrophobic pocket in which Y98 resides. **c**, Interactions made by S105 in domain I. **d**, Hydrophobic pocket formed by P261 and V262 at the interface between α8 and the β1-α8 loop. **e**, Polar interactions formed by E266 at the interface between α8 and the β1-α8 loop.

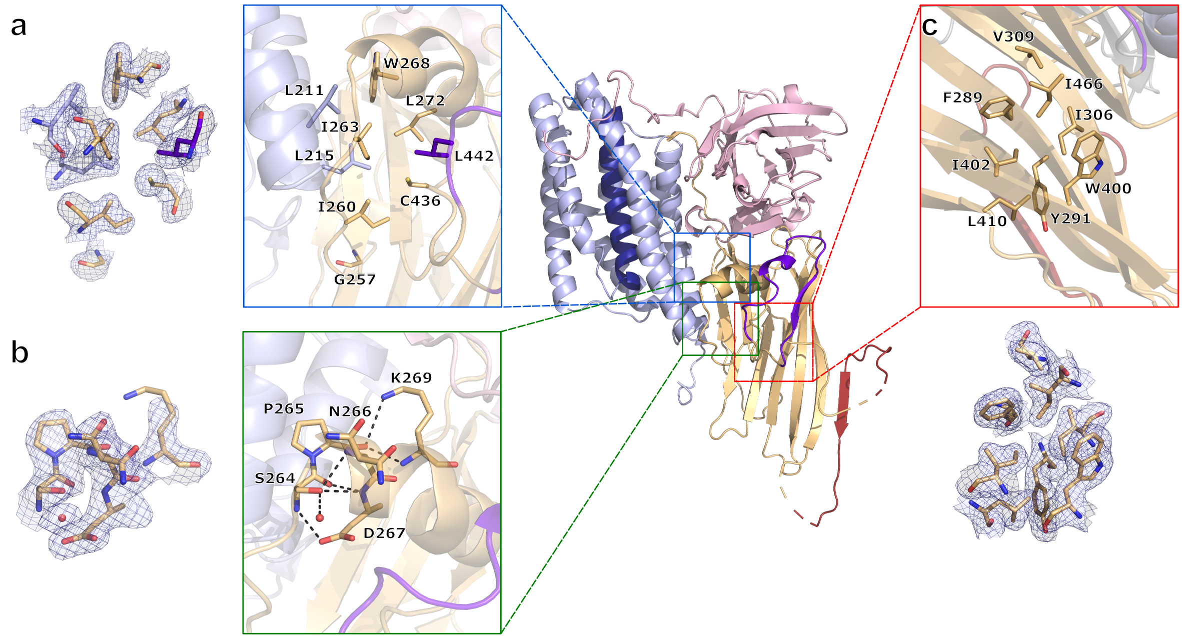

Supplementary Fig. 18. Structural interpretation of Cry11Ba mutations described in literature. Chain A of Cry11Ba is shown, colored as in Fig. 2. The $2F_{obs}$-F_{calc} electron density maps on the bottom of each panel are contoured at 1 σ. **a**, Hydrophobic pocket in which I263 resides and position of G257 in the turn between α8 and the β1-α8 loop. **b**, Polar interactions formed by S264 and K269 in the turn between α8 and the β1-α8 loop. **c**, Hydrophobic pocket in which I306 fits. Dashes indicate hydrogen bonds (up to 3.2 Å), and the salt bridge between K269(NZ) and P265(O) in (**b**).

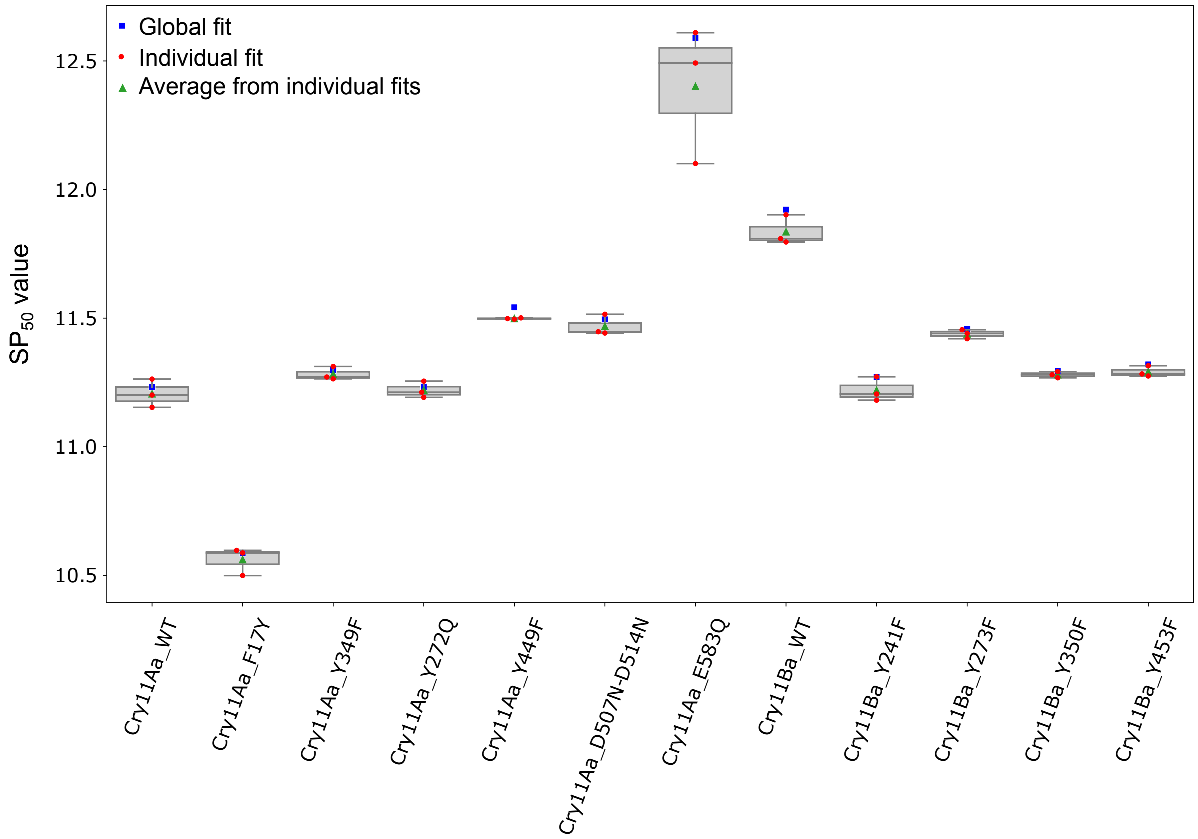

Supplementary Fig. 19. SP_{50} values determined for Cry11Aa, Cry11Ba and their mutants. Blue squares show the SP_{50} values as determined from a global fit to the measurements (n=3 individual experiments). Red spheres and boxplots show the SP_{50} values determined from fitting the three individual measurements, with green triangles showing the average value. The boxes represent the lower and upper quartiles around the median. Whiskers indicate the minimum and maximum values measured amongst replicates.

Supplementary Table 1. Interactions in the mutated interfaces. Hydrogen bonds up to 3.2 Å are listed.

a. Direct and single water-mediated interactions formed by D515, Y272 and D507

Interaction partner 1	Interaction partner 2	Distance (Å)*
D514 (OD2)	S249 (O)	2.9 / 2.9
	S251 (OG)	2.4
D514 (OD1)	S251 (N)	3.1
	W253 (NE1)	2.4 / 3.0
	Y272 (OH)	2.7
	R222 (NH1)	2.4 / 3.1
Y272 (OH)	R222 (NH1)	3.2 / 3.1
D507 (OD1)	R222 (NH1)	2.5
D507 (OD2)	R222 (NH2)	3.2
	Y203 (OH)	2.7 / 2.7
	W267 (NE1)	2.9

* Multiple distances indicate water-mediated hydrogen bonds.

b, Extended water-mediated interactions formed by E583 and other residues involved in this network

Interaction partner 1	Interaction partner 2	Distance (Å)*
E583 (OE1)	T197 (OG1)	2.8 / 2.9
	L193 (O)	2.8 / 2.8
	V192 (O)	2.6 / 2.7 / 3.0 / 3.1
	A233 (O)	2.6 / 2.7 / 2.8 / 2.9 / 2.6 / 2.9
W584 (N)	V192 (O)	3.2 / 2.7 / 3.0 / 3.1
	A223 (O)	3.2 / 2.7 / 2.8 / 2.9 / 2.6 / 2.9
Q511 (NE2)	V192 (O)	3.0 / 2.8 / 3.0 / 3.1
	A223 (O)	3.0 / 2.9 / 2.6 / 2.9
D510 (O)	V192 (O)	3.0 / 2.9 / 2.8 / 3.0 / 3.1
	A223 (O)	3.0 / 2.6 / 2.9
D510 (OD2)	R196 (NE)	3.1
	R196 (NH2)	3.3

* Multiple distances indicate water-mediated hydrogen bonds.

c, Extended water-mediated interactions formed by Y449 and D501, connecting two monomers via interface #1

Interaction partner monomer 1	Interaction partner monomer 2	Distance (Å)*
Y449 (OH)	V499 (N)	2.7 / 2.8
	V499 (O)	2.9 / 2.8
	T448 (OG1)	2.9 / 2.6
	D501 (OD1)	2.9 / 2.8
D501 (OD2)	Q281 (NE2)	2.8 / 2.9 / 2.6 / 3.0

* Multiple distances indicate water-mediated hydrogen bonds.

d, Hydrogen bonds made by Y349

Interaction partner monomer 1	Interaction partner monomer 2	Distance (Å)
Y349 (OH)	E295 (OE1)	3.1

Supplementary Table 2. Effects of Cry11Aa and Cry11Ba point mutations on toxicity described in literature

Mutant		Toxicity *Aedes aegypti*	*Anopheles stephensi*	*Culex quinquefasciatus*	References§
Cry11Aa	R90E	Not toxic	N.D.*	N.D.	1
	E97A	Not toxic	N.D.	N.D.	1, 2
	Y98E	Not toxic	N.D.	N.D.	1
	V104E	No crystal formation	No crystal formation	No crystal formation	1
	S105E	Not toxic	N.D.	N.D.	1
	V142D	Not toxic	N.D.	N.D.	2
	P261A	No change / 3-fold reduced	N.D.	N.D.	3, 4
	V262A	No change / 5-fold reduced	N.D.	N.D.	3, 4
	V262E	Not toxic	N.D.	N.D.	3
	E266A	No change / 21-fold reduced	N.D.	N.D.	3, 4
Cry11Ba	G257A	Strongly reduced	Reduced	Strongly reduced	5
	I263A	Not toxic	Not toxic	Strongly reduced	5
	S264A	Not toxic	No change	Reduced	5
	K269A	Strongly reduced	No change	Strongly reduced	5
	I306A	Not toxic	No change	Reduced	5

* N.D.: Not determined;

§ References:

1. Munoz-Garay, C. et al. Oligomerization of Cry11Aa from Bacillus thuringiensis Has an Important Role in Toxicity against Aedes aegypti. Appl. Environ. Microbiol. 75, 7548–7550 (2009).
2. Carmona, D. et al. Dominant Negative Phenotype of Bacillus thuringiensis Cry1Ab, Cry11Aa and Cry4Ba Mutants Suggest Hetero-Oligomer Formation among Different Cry Toxins. PLoS ONE 6, e19952 (2011).
3. Fernandez, L. E. et al. Cry11Aa toxin from Bacillus thuringiensis binds its receptor in Aedes aegypti mosquito larvae through loop alpha-8 of domain II. Febs Lett. 579, 3508–3514 (2005).
4. Perez, C. et al. Bacillus thuringiensis subsp israelensis Cyt1Aa synergizes Cry11Aa toxin by functioning as a membrane-bound receptor. Proc. Natl. Acad. Sci. U. S. A. 102, 18303–18308 (2005).
5. Likitvivatanavong, S., Aimanova, K. G. & Gill, S. S. Loop residues of the receptor binding domain of Bacillus thuringiensis Cry11Ba toxin are important for mosquitocidal activity. FEBS Lett. 583, 2021–2030 (2009).

Supplementary Table 3. Primers used to generate the Cry11Aa mutants.

Mutation	Forward / Reverse	Primer sequence (5'-3')	Comment
/	Forward	GCCGCAGTGTTATCACTCATGGTTATGGC	Amp_F1 was used with each reverse primer of Cry11Aa mutant construction. For each mutation primer, the inserted mutation is indicated as a capital bold letter. To generate the vector containing the mutation, the two fragments generated for the same mutations were assembled by Gibson assembly following the procedure described in the manuscript.
F17Y	Reverse	TTATATAATGGA**T**AGTCTGTTTCATTAACTATACTTAAAGTATCTAAAGAACTATCT	
Y272Q	Reverse	TAATAACTT**T**T**G**AACTAGTCCTCCCCATTCATTATAATTTACAGG	
Y349F	Reverse	TGGATTTTGA**A**AAAAAGTTTGTGTTCTAACTTCCATAATTT	
Y449F	Reverse	TCTATTCTATTA**A**ATGTTAGTGTTGTAAGATCGTGATTATATGCAAG	
D507N-D514N	Reverse	TTGCTTGATCTGGCGTAT**T**TTCTAAAAATGATCTATCT	
E583Q	Reverse	ATCCACCCATT**G**TGGATTAGCATTTG	
/	Reverse	AGTGCTGCCATAACCATGAGTGATAACACT	Amp_R1 was used with each forward primer of Cry11Aa mutant construction. For each mutation primer, the inserted mutation is indicated as a capital bold letter. To generate the vector containing the mutation, the two fragments generated for the same mutations were assembled by Gibson assembly following the procedure described in the manuscript.
F17Y	Forward	GTTAATGAAACAGACT**A**TCCATTATATAATAATTATACCGAACCTACT	
Y272Q	Forward	GGGAGGACTAGTT**C**A**A**AAGTTATTAATGGGGGA	
Y349F	Forward	CACAAACTTTTTT**T**TCAAAATCCAAATAATGAGCCT	
Y449F	Forward	TTACAACACTAACAT**T**TAATAGAATAGAGTATGATTCACCTACTACAG	
D507N-D514N	Forward	ATACGCCAGATCAAGCAACA**A**ACGGCAGTATTAAATTTG	
E583Q	Forward	TAATCCA**C**AATGGGTGGATTTTGTCACAG	
C11AB chimera	Forward	CTATCCTAAATAGGCGATCGCACTCATTAGGC	This fragment was obtained using Amp_R1 as reverse primer.
C11AB chimera	Reverse	CGTACAATAACCTTTAGTAACGGATTAATTTGCGTCGTAAAGG	This fragment was obtained using Amp_F1 as forward primer.
C11AB chimera	Forward	CGTTACTAAAGGTTATTGTACGTTGTCCGGATACTTTTTTTGTG	This fragment was assembled with the two above-mentioned fragments by Gibson to create the shuffle vector encoding the C11AB chimera.
C11AB chimera	Reverse	CGATCGCCTATTTAGGATAGTTATTGTTATACCCTTGGTTACATGTACAG	

Supplementary Table 4. Primers used to generate the Cry11Ba mutants.

Mutation	Gblock sequence
Silent WT mutants to reduce repeats and secondary structures	GACCATGATTACGAATTGGTACCTTTTCGATTTCAAATTTTCCAAACTTAAATATGATTGAATGCCTGAGA<br>AAGGTAATAGAGATGTTTTAGTTTATTATGAAGTATTAGGGGCGTCTTTTAAATTCAATCTATCAATTTGT<br>GAAATATATTACTCAAAACCCAATACCATTCTAAAACTTATTCAAAATATATATTGCTTTAAAAGAGCATA<br>CATACTAAAAAAACAGGCATCTTTCGAACTATAGCGCATAGAATACTACGGTGAATCAAAAACAAATAAA<br>ATTTAGGAGGTATATTCAAGTATACAAAAAAACTTTAGTGTGAGGGGATTTAGATAAAAAGTATTCGTTAT<br>CCTTATAAATTAATTCTTAAACATGCACCAATGTATACATTAAATAATATTATGTGAATTAAGTCTATCAAT<br>TTAATTTATTATGTTACTTTATATTTGATTAATAATTGCAAGTTTAAAATCATAATTTAATGTTGAAAGGCC<br>ACTATTCTAATTAACTTAAGGAGTTGTTTATTTATGCAAAATAACAACTTTAATACCACAGAAATTAATAAT<br>ATGATTAATTTCCCTATGTATAATGGTAGATTAGAACCTTCTCTAGCTCCAGCATTAATAGCAGTAGCTC<br>CAATTGCTAAATATTTAGCAACAGCTCTTGCTAAATGGGCTGTAAAACAAGGGTTTGCAAAATTAAAATC<br>CGAGATATTCCCCGGTAATACGCCTGCTACTATGGATAAGGTTCGTATTGAGGTACAAACACTTTTAGA<br>CCAAAGATTACAAGATGACAGAGTTAAGATTTTAGAAGGTGAATACAAAGGAATTATTGACGTGAGTAAA<br>GTTTTTACTGATTATGTTAATCAATCTAAATTTGAGACTGGAACAGCTAATAGGCTTTTTTTTGATACAAG<br>TAACCAATTAATAAGCAGATTGCCTCAATTTGAGATTGCAGGATATGAAGGAGTATCCATTTCACTTTTT<br>ACTCAGATGTGTACATTTCATTTGGGTTTATTAAAAGATGGAATTTTAGCAGGAAGCGATTGGGGATTTG<br>CTCCTGCAGATAAAGACGCTCTTATTTGCCAATTTAATAGATTTGTCAATGAATATAATACTCGACTGAT<br>GGTATTGTACTCAAAAGAATTTGGACGGTTATTAGCAAAAAATCTTAATGAAGCCTTGAACTTTAGAAAT<br>ATGTGTAGTTTATATGTCTTTCCTTTTTCTGAAGCATGGTCTTTATTAAGGTATGAAGGAACAAAATTAGA<br>AAACACGCTTTCATTATGGAATTTTGTGGGTGAAAGTATCAATAATATATCTCCTAATGATTGGAAAGGT<br>GCGCTTTATAAATTGTTAATGGGAGCACCTAATCAAAGATTAAACAATGTTAAGTTTAATTATAGTTATTT<br>TTCTGATACTCAAGCGACAATACATCGTGAAAACATTCATGGTGTCCTGCCAACATATAATGGAGGACC<br>AACAATTACAGGATGGATAGGGAATGGGCGTTTCAGCGGACTTAGTTTTCCTTGTAGTAATGAATTAGA<br>AATTACAAAAATAAAACAGGAAATAACTTACAATGATAAAGGGGGAAATTTCAATTCAATAGTTCCTGCT<br>GCTACGCGCAATGAAATTCTAACTGCTACCGTTCCAACATCAGCTGATCCATTTTTTAAAACCGCTGATA<br>TTAACTGGAAATATTTCTCTCCGGGTCTTTACTCTGGATGGAATATTAAATTTGATGATACAGTCACTTTA<br>AAAAGTAGAGTACCAAGTATTATACCTTCAAATATATTAAAGTATGATGATTATTATATTCGTGCCGTTTC<br>AGCCTGTCCAAAAGGCGTATCACTTGCATATAACCATGATTTTTTAACGTTAACATATAACAAATTAGAAT<br>ATGATGCACCTACTACACAAAATATCATTGTAGGATTTTCACCAGATAATACTAAGAGTTTTTATAGGAG<br>CAACTCTCATTATCTAAGTACAACAGATGATGCCTATGTAATTCCTGCTTTACAATTTTCTACAGTCTCAG<br>ATAGATCATTCTTAGAAGATACACCAGATCAAGCAACAGATGGCAGTATTAAATTTACGGATACTGTTCT<br>TGGGAATGAGGCAAAATATTCTATTAGACTAAATACTGGATTTAATACAGCTACTAGGTATAGATTAATTA<br>TACGTTTTAAAGCGCCTGCTCGTTTGGCTGCTGGTATACGTGTACGTTCTCAAAATTCAGGGAATAATAA<br>GTTATTAGGTGGTATTCCTGTAGAGGGTAATTCTGGATGGATAGATTATATTACAGATTCATTTACTTTTG<br>ATGACCTTGGGATTACAACTTCAAGTACAAATGCTTTCTTTAGTATTGATTCAGATGGTGTAAATGCTTCT<br>CAACAATGGTATTTGTCTAAATTAATTTTAGTAAAAGAATCCAGTTTTACGACTCAGATTCCATTAAAACC<br>ATACGTTATTGTACGTTGTCCGGATACTTTTTTTGTGAGCAACAATTCAAGTAGTACGTACGAACAAGGC<br>TATAACAACAATTACAACCAGAATTCTAGCAGTATGTACGATCAAGGATATAACAATAGCTATAATCCAA<br>ACTCTGGTTGTACGTGTAATCAAGACTATAATAACAGTTATAACCAAAACTCTGGCTGTACATGTAACCA<br>AGGGTATAACAATAACTATCCTAAATAATCTTAGTAGCTATATTTATTAAATATGGTAATATCACAAGTAT<br>AAATACTTGTGGTATTACCTACCATTCTTAAATTATATCCAAAATCATGCGTTAATCTACATTCCCCTTTC<br>TCTAAAATTTGTTCTTCACACATCCACATTTTTCGACTCGAGGCATGCAAGCTTGGC

(Continued)

Mutation	Gblock sequence*
Y241F	GACCATGATTACGAATTGGTACCTTTTCGATTTCAAATTTTCCAAACTTAAATATGATTGAATGCCTGAGA<br>AAGGTAATAGAGATGTTTTAGTTTATTATGAAGTATTAGGGGCGTCTTTTAAATTCAATCTATCAATTTGT<br>GAAATATATTACTCAAAACCCAATACCATTCTAAAACTTATTCAAAATATATATTGCTTTAAAAGAGCATA<br>CATACTAAAAAAACAGGCATCTTTCGAACTATAGCGCATAGAATACTACGGTGAATCAAAAACAAATAAA<br>ATTTAGGAGGTATATTCAAGTATACAAAAAAACTTTAGTGTGAGGGGATTTAGATAAAAAGTATTCGTTAT<br>CCTTATAAATTAATTCTTAAACATGCACCAATGTATACATTAAATAATATTATGTGAATTAAGTCTATCAAT<br>TTAATTTATTATGTTACTTTATATTTGATTAATAATTGCAAGTTTAAAATCATAATTTAATGTTGAAAGGCC<br>ACTATTCTAATTAACTTAAGGAGTTGTTTATTTATGCAAAATAACAACTTTAATACCACAGAAATTAATAAT<br>ATGATTAATTTCCCTATGTATAATGGTAGATTAGAACCTTCTCTAGCTCCAGCATTAATAGCAGTAGCTC<br>CAATTGCTAAATATTTAGCAACAGCTCTTGCTAAATGGGCTGTAAAACAAGGGTTTGCAAAATTAAAATC<br>CGAGATATTCCCCGGTAATACGCCTGCTACTATGGATAAGGTTCGTATTGAGGTACAAACACTTTTAGA<br>CCAAAGATTACAAGATGACAGAGTTAAGATTTTAGAAGGTGAATACAAAGGAATTATTGACGTGAGTAAA<br>GTTTTTACTGATTATGTTAATCAATCTAAATTTGAGACTGGAACAGCTAATAGGCTTTTTTTTGATACAAG<br>TAACCAATTAATAAGCAGATTGCCTCAATTTGAGATTGCAGGATATGAAGGAGTATCCATTTCACTTTTT<br>ACTCAGATGTGTACATTTCATTTGGGTTTATTAAAAGATGGAATTTTAGCAGGAAGCGATTGGGGATTTG<br>CTCCTGCAGATAAAGACGCTCTTATTTGCCAATTTAATAGATTTGTCAATGAATATAATACTCGACTGAT<br>GGTATTGTACTCAAAAGAATTTGGACGGTTATTAGCAAAAAATCTTAATGAAGCCTTGAACTTTAGAAAT<br>ATGTGTAGTTTATATGTCTTTCCTTTTTCTGAAGCATGGTCTTTATTAAGGT**T**TGAAGGAACAAAATTAGA<br>AAACACGCTTTCATTATGGAATTTTGTGGGTGAAAGTATCAATAATATATCTCCTAATGATTGGAAAGGT<br>GCGCTTTATAAATTGTTAATGGGAGCACCTAATCAAAGATTAAACAATGTTAAGTTTAATTATAGTTATTT<br>TTCTGATACTCAAGCGACAATACATCGTGAAAACATTCATGGTGTCCTGCCAACATATAATGGAGGACC<br>AACAATTACAGGATGGATAGGGAATGGGCGTTTCAGCGGACTTAGTTTTCCTTGTAGTAATGAATTAGA<br>AATTACAAAAATAAAACAGGAAATAACTTACAATGATAAAGGGGGAAATTTCAATTCAATAGTTCCTGCT<br>GCTACGCGCAATGAAATTCTAACTGCTACCGTTCCAACATCAGCTGATCCATTTTTTAAAACCGCTGATA<br>TTAACTGGAAATATTTCTCTCCGGGTCTTTACTCTGGATGGAATATTAAATTTGATGATACAGTCACTTTA<br>AAAAGTAGAGTACCAAGTATTATACCTTCAAATATATTAAAGTATGATGATTATTATATTCGTGCCGTTTC<br>AGCCTGTCCAAAAGGCGTATCACTTGCATATAACCATGATTTTTTAACGTTAACATATAACAAATTAGAAT<br>ATGATGCACCTACTACACAAAATATCATTGTAGGATTTTCACCAGATAATACTAAGAGTTTTTATAGGAG<br>CAACTCTCATTATCTAAGTACAACAGATGATGCCTATGTAATTCCTGCTTTACAATTTTCTACAGTCTCAG<br>ATAGATCATTCTTAGAAGATACACCAGATCAAGCAACAGATGGCAGTATTAAATTTACGGATACTGTTCT<br>TGGGAATGAGGCAAAATATTCTATTAGACTAAATACTGGATTTAATACAGCTACTAGGTATAGATTAATTA<br>TACGTTTTAAAGCGCCTGCTCGTTTGGCTGCTGGTATACGTGTACGTTCTCAAAATTCAGGGAATAATAA<br>GTTATTAGGTGGTATTCCTGTAGAGGGTAATTCTGGATGGATAGATTATATTACAGATTCATTTACTTTTG<br>ATGACCTTGGGATTACAACTTCAAGTACAAATGCTTTCTTTAGTATTGATTCAGATGGTGTAAATGCTTCT<br>CAACAATGGTATTTGTCTAAATTAATTTTAGTAAAAGAATCCAGTTTTACGACTCAGATTCCATTAAAACC<br>ATACGTTATTGTACGTTGTCCGGATACTTTTTTTGTGAGCAACAATTCAAGTAGTACGTACGAACAAGGC<br>TATAACAACAATTACAACCAGAATTCTAGCAGTATGTACGATCAAGGATATAACAATAGCTATAATCCAA<br>ACTCTGGTTGTACGTGTAATCAAGACTATAATAACAGTTATAACCAAAACTCTGGCTGTACATGTAACCA<br>AGGGTATAACAATAACTATCCTAAATAATCTTAGTAGCTATATTTATTAAATATGGTAATATCACAAGTAT<br>AAATACTTGTGGTATTACCTACCATTCTTAAATTATATCCAAAATCATGCGTTAATCTACATTCCCCTTTC<br>TCTAAAATTTGTTCTTCACACATCCACATTTTTCGACTCGAGGCATGCAAGCTTGGC

*The inserted mutation is indicated as a capital bold letter and underlined

(Continued)

Mutation	Gblock sequence*
Y273F	GACCATGATTACGAATTGGTACCTTTTCGATTTCAAATTTTCCAAACTTAAATATGATTGAATGCCTGAGA<br>AAGGTAATAGAGATGTTTTAGTTTATTATGAAGTATTAGGGGCGTCTTTTAAATTCAATCTATCAATTTGT<br>GAAATATATTACTCAAAACCCAATACCATTCTAAAACTTATTCAAAATATATATTGCTTTAAAAGAGCATA<br>CATACTAAAAAAACAGGCATCTTTCGAACTATAGCGCATAGAATACTACGGTGAATCAAAAACAAATAAA<br>ATTTAGGAGGTATATTCAAGTATACAAAAAAACTTTAGTGTGAGGGGATTTAGATAAAAAGTATTCGTTAT<br>CCTTATAAATTAATTCTTAAACATGCACCAATGTATACATTAAATAATATTATGTGAATTAAGTCTATCAAT<br>TTAATTTATTATGTTACTTTATATTTGATTAATAATTGCAAGTTTAAAATCATAATTTAATGTTGAAAGGCC<br>ACTATTCTAATTAACTTAAGGAGTTGTTTATTTATGCAAAATAACAACTTTAATACCACAGAAATTAATAAT<br>ATGATTAATTTCCCTATGTATAATGGTAGATTAGAACCTTCTCTAGCTCCAGCATTAATAGCAGTAGCTC<br>CAATTGCTAAATATTTAGCAACAGCTCTTGCTAAATGGGCTGTAAAACAAGGGTTTGCAAAATTAAAATC<br>CGAGATATTCCCCGGTAATACGCCTGCTACTATGGATAAGGTTCGTATTGAGGTACAAACACTTTTAGA<br>CCAAAGATTACAAGATGACAGAGTTAAGATTTTAGAAGGTGAATACAAAGGAATTATTGACGTGAGTAAA<br>GTTTTTACTGATTATGTTAATCAATCTAAATTTGAGACTGGAACAGCTAATAGGCTTTTTTTTGATACAAG<br>TAACCAATTAATAAGCAGATTGCCTCAATTTGAGATTGCAGGATATGAAGGAGTATCCATTTCACTTTTT<br>ACTCAGATGTGTACATTTCATTTGGGTTTATTAAAAGATGGAATTTTAGCAGGAAGCGATTGGGGATTTG<br>CTCCTGCAGATAAAGACGCTCTTATTTGCCAATTTAATAGATTTGTCAATGAATATAATACTCGACTGAT<br>GGTATTGTACTCAAAAGAATTTGGACGGTTATTAGCAAAAAATCTTAATGAAGCCTTGAACTTTAGAAAT<br>ATGTGTAGTTTATATGTCTTTCCTTTTTCTGAAGCATGGTCTTTATTAAGGTATGAAGGAACAAAATTAGA<br>AAACACGCTTTCATTATGGAATTTTGTGGGTGAAAGTATCAATAATATATCTCCTAATGATTGGAAAGGT<br>GCGCTTT**T**TAAATTGTTAATGGGAGCACCTAATCAAAGATTAAACAATGTTAAGTTTAATTATAGTTATTT<br>TTCTGATACTCAAGCGACAATACATCGTGAAAACATTCATGGTGTCCTGCCAACATATAATGGAGGACC<br>AACAATTACAGGATGGATAGGGAATGGGCGTTTCAGCGGACTTAGTTTTCCTTGTAGTAATGAATTAGA<br>AATTACAAAAATAAAACAGGAAATAACTTACAATGATAAAGGGGGAAATTTCAATTCAATAGTTCCTGCT<br>GCTACGCGCAATGAAATTCTAACTGCTACCGTTCCAACATCAGCTGATCCATTTTTTAAAACCGCTGATA<br>TTAACTGGAAATATTTCTCTCCGGGTCTTTACTCTGGATGGAATATTAAATTTGATGATACAGTCACTTTA<br>AAAAGTAGAGTACCAAGTATTATACCTTCAAATATATTAAAGTATGATGATTATTATATTCGTGCCGTTTC<br>AGCCTGTCCAAAAGGCGTATCACTTGCATATAACCATGATTTTTTAACGTTAACATATAACAAATTAGAAT<br>ATGATGCACCTACTACACAAAATATCATTGTAGGATTTTCACCAGATAATACTAAGAGTTTTTATAGGAG<br>CAACTCTCATTATCTAAGTACAACAGATGATGCCTATGTAATTCCTGCTTTACAATTTTCTACAGTCTCAG<br>ATAGATCATTCTTAGAAGATACACCAGATCAAGCAACAGATGGCAGTATTAAATTTACGGATACTGTTCT<br>TGGGAATGAGGCAAAATATTCTATTAGACTAAATACTGGATTTAATACAGCTACTAGGTATAGATTAATTA<br>TACGTTTTAAAGCGCCTGCTCGTTTGGCTGCTGGTATACGTGTACGTTCTCAAAATTCAGGGAATAATAA<br>GTTATTAGGTGGTATTCCTGTAGAGGGTAATTCTGGATGGATAGATTATATTACAGATTCATTTACTTTTG<br>ATGACCTTGGGATTACAACTTCAAGTACAAATGCTTTCTTTAGTATTGATTCAGATGGTGTAAATGCTTCT<br>CAACAATGGTATTTGTCTAAATTAATTTTAGTAAAAGAATCCAGTTTTACGACTCAGATTCCATTAAAACC<br>ATACGTTATTGTACGTTGTCCGGATACTTTTTTTGTGAGCAACAATTCAAGTAGTACGTACGAACAAGGC<br>TATAACAACAATTACAACCAGAATTCTAGCAGTATGTACGATCAAGGATATAACAATAGCTATAATCCAA<br>ACTCTGGTTGTACGTGTAATCAAGACTATAATAACAGTTATAACCAAAACTCTGGCTGTACATGTAACCA<br>AGGGTATAACAATAACTATCCTAAATAATCTTAGTAGCTATATTTATTAAATATGGTAATATCACAAGTAT<br>AAATACTTGTGGTATTACCTACCATTCTTAAATTATATCCAAAATCATGCGTTAATCTACATTCCCCTTTC<br>TCTAAAATTTGTTCTTCACACATCCACATTTTTCGACTCGAGGCATGCAAGCTTGGC

*The inserted mutation is indicated as a capital bold letter and underlined.

(Continued)

Mutation	Gblock sequence*
Y350F	GACCATGATTACGAATTGGTACCTTTTCGATTTCAAATTTTCCAAACTTAAATATGATTGAATGCCTGAGA<br>AAGGTAATAGAGATGTTTTAGTTTATTATGAAGTATTAGGGGCGTCTTTTAAATTCAATCTATCAATTTGT<br>GAAATATATTACTCAAAACCCAATACCATTCTAAAACTTATTCAAAATATATATTGCTTAAAAGAGCATA<br>CATACTAAAAAAACAGGCATCTTTCGAACTATAGCGCATAGAATACTACGGTGAATCAAAAACAAATAAA<br>ATTTAGGAGGTATATTCAAGTATACAAAAAAACTTTAGTGTGAGGGGATTTAGATAAAAAGTATTCGTTAT<br>CCTTATAAATTAATTCTTAAACATGCACCAATGTATACATTAAATAATATTATGTGAATTAAGTCTATCAAT<br>TTAATTTATTATGTTACTTTATATTTGATTAATAATTGCAAGTTTAAAATCATAATTTAATGTTGAAAGGCC<br>ACTATTCTAATTAACTTAAGGAGTTGTTTATTTATGCAAAATAACAACTTTAATACCACAGAAATTAATAAT<br>ATGATTAATTTCCCTATGTATAATGGTAGATTAGAACCTTCTCTAGCTCCAGCATTAATAGCAGTAGCTC<br>CAATTGCTAAATATTTAGCAACAGCTCTTGCTAAATGGGCTGTAAAACAAGGGTTTGCAAAATTAAAATC<br>CGAGATATTCCCCGGTAATACGCCTGCTACTATGGATAAGGTTCGTATTGAGGTACAAACACTTTTAGA<br>CCAAAGATTACAAGATGACAGAGTTAAGATTTTAGAAGGTGAATACAAAGGAATTATTGACGTGAGTAAA<br>GTTTTTACTGATTATGTTAATCAATCTAAATTTGAGACTGGAACAGCTAATAGGCTTTTTTTTGATACAAG<br>TAACCAATTAATAAGCAGATTGCCTCAATTTGAGATTGCAGGATATGAAGGAGTATCCATTTCACTTTTT<br>ACTCAGATGTGTACATTTCATTTGGGTTTATTAAAAGATGGAATTTTAGCAGGAAGCGATTGGGGATTTG<br>CTCCTGCAGATAAAGACGCTCTTATTTGCCAATTTAATAGATTTGTCAATGAATATAATACTCGACTGAT<br>GGTATTGTACTCAAAAGAATTTGGACGGTTATTAGCAAAAAATCTTAATGAAGCCTTGAACTTTAGAAAT<br>ATGTGTAGTTTATATGTCTTTCCTTTTTCTGAAGCATGGTCTTTATTAAGGTATGAAGGAACAAAATTAGA<br>AAACACGCTTTCATTATGGAATTTTGTGGGTGAAAGTATCAATAATATATCTCCTAATGATTGGAAAGGT<br>GCGCTTTATAAATTGTTAATGGGAGCACCTAATCAAAGATTAAACAATGTTAAGTTTAATTATAGTTATTT<br>TTCTGATACTCAAGCGACAATACATCGTGAAAACATTCATGGTGTCCTGCCAACATATAATGGAGGACC<br>AACAATTACAGGATGGATAGGGAATGGGCGTTTCAGCGGACTTAGTTTTCCTTGTAGTAATGAATTAGA<br>AATTACAAAAATAAAACAGGAAATAACTT**T**CAATGATAAAGGGGGAAATTTCAATTCAATAGTTCCTGCT<br>GCTACGCGCAATGAAATTCTAACTGCTACCGTTCCAACATCAGCTGATCCATTTTTTAAAACCGCTGATA<br>TTAACTGGAAATATTTCTCTCCGGGTCTTTACTCTGGATGGAATATTAAATTTGATGATACAGTCACTTTA<br>AAAAGTAGAGTACCAAGTATTATACCTTCAAATATATTAAAGTATGATGATTATTATATTCGTGCCGTTTC<br>AGCCTGTCCAAAAGGCGTATCACTTGCATATAACCATGATTTTTTAACGTTAACATATAACAAATTAGAAT<br>ATGATGCACCTACTACACAAAATATCATTGTAGGATTTTCACCAGATAATACTAAGAGTTTTTATAGGAG<br>CAACTCTCATTATCTAAGTACAACAGATGATGCCTATGTAATTCCTGCTTTACAATTTTCTACAGTCTCAG<br>ATAGATCATTCTTAGAAGATACACCAGATCAAGCAACAGATGGCAGTATTAAATTTACGGATACTGTTCT<br>TGGGAATGAGGCAAAATATTCTATTAGACTAAATACTGGATTTAATACAGCTACTAGGTATAGATTAATTA<br>TACGTTTTAAAGCGCCTGCTCGTTTGGCTGCTGGTATACGTGTACGTTCTCAAAATTCAGGGAATAATAA<br>GTTATTAGGTGGTATTCCTGTAGAGGGTAATTCTGGATGGATAGATTATATTACAGATTCATTTACTTTTG<br>ATGACCTTGGGATTACAACTTCAAGTACAAATGCTTTCTTTAGTATTGATTCAGATGGTGTAAATGCTTCT<br>CAACAATGGTATTTGTCTAAATTAATTTTAGTAAAAGAATCCAGTTTTACGACTCAGATTCCATTAAAACC<br>ATACGTTATTGTACGTTGTCCGGATACTTTTTTTGTGAGCAACAATTCAAGTAGTACGTACGAACAAGGC<br>TATAACAACAATTACAACCAGAATTCTAGCAGTATGTACGATCAAGGATATAACAATAGCTATAATCCAA<br>ACTCTGGTTGTACGTGTAATCAAGACTATAATAACAGTTATAACCAAAACTCTGGCTGTACATGTAACCA<br>AGGGTATAACAATAACTATCCTAAATAATCTTAGTAGCTATATTTATTAAATATGGTAATATCACAAGTAT<br>AAATACTTGTGGTATTACCTACCATTCTTAAATTATATCCAAAATCATGCGTTAATCTACATTCCCCTTTC<br>TCTAAAATTTGTTCTTCACACATCCACATTTTTCGACTCGAGGCATGCAAGCTTGGC

*The inserted mutation is indicated as a capital bold letter and underlined.

(Continued)

Mutation	Gblock sequence*
Y453F	GACCATGATTACGAATTGGTACCTTTTCGATTTCAAATTTTCCAAACTTAAATATGATTGAATGCCTGAGA<br>AAGGTAATAGAGATGTTTTAGTTTATTATGAAGTATTAGGGGCGTCTTTTAAATTCAATCTATCAATTTGT<br>GAAATATATTACTCAAAACCCAATACCATTCTAAAACTTATTCAAAATATATATTGCTTAAAAGAGCATA<br>CATACTAAAAAAACAGGCATCTTTCGAACTATAGCGCATAGAATACTACGGTGAATCAAAAACAAATAAA<br>ATTTAGGAGGTATATTCAAGTATACAAAAAAACTTTAGTGTGAGGGGATTTAGATAAAAAGTATTCGTTAT<br>CCTTATAAATTAATTCTTAAACATGCACCAATGTATACATTAAATAATATTATGTGAATTAAGTCTATCAAT<br>TTAATTTATTATGTTACTTTATATTTGATTAATAATTGCAAGTTTAAAATCATAATTTAATGTTGAAAGGCC<br>ACTATTCTAATTAACTTAAGGAGTTGTTTATTTATGCAAAATAACAACTTTAATACCACAGAAATTAATAAT<br>ATGATTAATTTCCCTATGTATAATGGTAGATTAGAACCTTCTCTAGCTCCAGCATTAATAGCAGTAGCTC<br>CAATTGCTAAATATTTAGCAACAGCTCTTGCTAAATGGGCTGTAAAACAAGGGTTTGCAAAATTAAAATC<br>CGAGATATTCCCCGGTAATACGCCTGCTACTATGGATAAGGTTCGTATTGAGGTACAAACACTTTTAGA<br>CCAAAGATTACAAGATGACAGAGTTAAGATTTTAGAAGGTGAATACAAAGGAATTATTGACGTGAGTAAA<br>GTTTTTACTGATTATGTTAATCAATCTAAATTTGAGACTGGAACAGCTAATAGGCTTTTTTTTGATACAAG<br>TAACCAATTAATAAGCAGATTGCCTCAATTTGAGATTGCAGGATATGAAGGAGTATCCATTTCACTTTTT<br>ACTCAGATGTGTACATTTCATTTGGGTTTATTAAAAGATGGAATTTTAGCAGGAAGCGATTGGGGATTTG<br>CTCCTGCAGATAAAGACGCTCTTATTTGCCAATTTAATAGATTTGTCAATGAATATAATACTCGACTGAT<br>GGTATTGTACTCAAAAGAATTTGGACGGTTATTAGCAAAAAATCTTAATGAAGCCTTGAACTTTAGAAAT<br>ATGTGTAGTTTATATGTCTTTCCTTTTTCTGAAGCATGGTCTTTATTAAGGTATGAAGGAACAAAATTAGA<br>AAACACGCTTTCATTATGGAATTTTGTGGGTGAAAGTATCAATAATATATCTCCTAATGATTGGAAAGGT<br>GCGCTTTATAAATTGTTAATGGGAGCACCTAATCAAAGATTAAACAATGTTAAGTTTAATTATAGTTATTT<br>TTCTGATACTCAAGCGACAATACATCGTGAAAACATTCATGGTGTCCTGCCAACATATAATGGAGGACC<br>AACAATTACAGGATGGATAGGGAATGGGCGTTTCAGCGGACTTAGTTTTCCTTGTAGTAATGAATTAGA<br>AATTACAAAAATAAAACAGGAAATAACTTACAATGATAAAGGGGGAAATTTCAATTCAATAGTTCCTGCT<br>GCTACGCGCAATGAAATTCTAACTGCTACCGTTCCAACATCAGCTGATCCATTTTTTAAAACCGCTGATA<br>TTAACTGGAAATATTTCTCTCCGGGTCTTTACTCTGGATGGAATATTAAATTTGATGATACAGTCACTTTA<br>AAAAGTAGAGTACCAAGTATTATACCTTCAAATATATTAAAGTATGATGATTATTATATTCGTGCCGTTTC<br>AGCCTGTCCAAAAGGCGTATCACTTGCATATAACCATGATTTTTTAACGTTAACAT**T**TAACAAATTAGAAT<br>ATGATGCACCTACTACACAAAATATCATTGTAGGATTTTCACCAGATAATACTAAGAGTTTTTATAGGAG<br>CAACTCTCATTATCTAAGTACAACAGATGATGCCTATGTAATTCCTGCTTTACAATTTTCTACAGTCTCAG<br>ATAGATCATTCTTAGAAGATACACCAGATCAAGCAACAGATGGCAGTATTAAATTTACGGATACTGTTCT<br>TGGGAATGAGGCAAAATATTCTATTAGACTAAATACTGGATTTAATACAGCTACTAGGTATAGATTAATTA<br>TACGTTTTAAAGCGCCTGCTCGTTTGGCTGCTGGTATACGTGTACGTTCTCAAAATTCAGGGAATAATAA<br>GTTATTAGGTGGTATTCCTGTAGAGGGTAATTCTGGATGGATAGATTATATTACAGATTCATTTACTTTTG<br>ATGACCTTGGGATTACAACTTCAAGTACAAATGCTTTCTTTAGTATTGATTCAGATGGTGTAAATGCTTCT<br>CAACAATGGTATTTGTCTAAATTAATTTTAGTAAAAGAATCCAGTTTTACGACTCAGATTCCATTAAAACC<br>ATACGTTATTGTACGTTGTCCGGATACTTTTTTTGTGAGCAACAATTCAAGTAGTACGTACGAACAAGGC<br>TATAACAACAATTACAACCAGAATTCTAGCAGTATGTACGATCAAGGATATAACAATAGCTATAATCCAA<br>ACTCTGGTTGTACGTGTAATCAAGACTATAATAACAGTTATAACCAAAACTCTGGCTGTACATGTAACCA<br>AGGGTATAACAATAACTATCCTAAATAATCTTAGTAGCTATATTTATTAAATATGGTAATATCACAAGTAT<br>AAATACTTGTGGTATTACCTACCATTCTTAAATTATATCCAAAATCATGCGTTAATCTACATTCCCCTTTC<br>TCTAAAATTTGTTCTTCACACATCCACATTTTTCGACTCGAGGCATGCAAGCTTGGC

*The inserted mutation is indicated as a capital bold letter and underlined.
