# Supplementary material for: De novo determination of mosquitocidal Cry11Aa and Cry11Ba structures from naturally-occurring nanocrystals
Source: Nat Commun. 2022 Jul 28;13:4376. doi: 10.1038/s41467-022-31746-x (PMC9334358; doi:10.1038/s41467-022-31746-x)
Supplement: Supplementary file 2 — Reporting Summary [file 41467_2022_31746_MOESM2_ESM.pdf]

nature portfolio

Corresponding author(s): Jacques-Philippe Colletier

Last updated by author(s): May 20, 2022

Reporting Summary

Nature Portfolio wishes to improve the reproducibility of the work that we publish. This form provides structure for consistency and transparency in reporting. For further information on Nature Portfolio policies, see our Editorial Policies and the Editorial Policy Checklist.

Statistics

For all statistical analyses, confirm that the following items are present in the figure legend, table legend, main text, or Methods section.

n/a	Confirmed	
☐	☒	The exact sample size (*n*) for each experimental group/condition, given as a discrete number and unit of measurement
☐	☒	A statement on whether measurements were taken from distinct samples or whether the same sample was measured repeatedly
☐	☒	The statistical test(s) used AND whether they are one- or two-sided<br>*Only common tests should be described solely by name; describe more complex techniques in the Methods section.*
☒	☐	A description of all covariates tested
☐	☒	A description of any assumptions or corrections, such as tests of normality and adjustment for multiple comparisons
☐	☒	A full description of the statistical parameters including central tendency (e.g. means) or other basic estimates (e.g. regression coefficient) AND variation (e.g. standard deviation) or associated estimates of uncertainty (e.g. confidence intervals)
☒	☐	For null hypothesis testing, the test statistic (e.g. *F*, *t*, *r*) with confidence intervals, effect sizes, degrees of freedom and *P* value noted<br>*Give P values as exact values whenever suitable.*
☒	☐	For Bayesian analysis, information on the choice of priors and Markov chain Monte Carlo settings
☒	☐	For hierarchical and complex designs, identification of the appropriate level for tests and full reporting of outcomes
☐	☒	Estimates of effect sizes (e.g. Cohen's *d*, Pearson's *r*), indicating how they were calculated

Our web collection on statistics for biologists contains articles on many of the points above.

Software and code

Policy information about availability of computer code

Data collection	Scanning electron micrographs (SEM) were acquired using a Zeiss LEO 1530 microscope and the associated commercial software. Transmission electron microscopy (TEM) images were collected on a Thermofisher TF20 electron microscope and its associated software. Atomic force microscopy (AFM) data was acquired on a Multimode 8, Nanoscope V (Bruker) controlled by the NanoScope software (Bruker, Santa Barbara, CA). X-ray diffraction data was collected at the Linac Coherent Light Source (LCLS), Stanford (USA) and EuXFEL, Hamburg (Germany), using the beamline's data collection software. Protein concentrations were measured on a Nanodrop 2000 or Nanodrop one (Thermo Fisher). Turbidity measurements were carried out on a NEPHELOstar Plus (BMG Labtech) nephelometer. SDS-PAGE gels were digitalized using a ChemiDoc XRS+ imaging system controlled by Image Lab software version 6.0.0 (BioRad, France). MALDI (TOF) mass spectra on Cry11Aa and Cry11Ba were acquired on a Bruker Autoflex and Bruker Rapiflex mass spectrometer (Bruker Daltonics, Bremen, Germany) and associated software, respectively. Gel Liquid Chromatography tandem mass spectrometry (GeLC-MS/MS) spectra collected on Cry11Ba were acquired on a ThermoFisher Q-Exactive Plus. Native mass spectrometry on Cry11Aa was collected on a Synapt G1 mass spectrometer (Waters Corporation). Thermal unfolding of Cry11Aa and Cry11Aa mutants was followed on a Prometheus NT.48 (Nanotemper).
Data analysis	AFM Images were processed with Gwyddion, and when needed stripe noise was removed using DeStripe. ImageJ v1.51k was used to analyse SEM images and SDS-PAGE gels. Low complexity region (LCR) of Cry11Ba was analysed using the LCR eXXXplorer. X-ray diffraction images were analyzed using NanoPeakCell and processed using CrystFEL (Cry11Aa and Cry11Aa mutants) or cctbx.xfel and PRIME (Cry11Ba). Experimental phasing was carried out using Crank2, while molecular replacement was performed using PhaserMr. All structures were refined in phenix.refine and coot. Structures were visually analyzed in Pymol. Structural interfaces were analyzed in PISA. Sequence alignments were carried out in strap. The AlphaFold2 and RosettaFold online servers were used for structure prediction. MALDI (TOF) data was analyzed using Flexanalysis v.3.0 (Bruker Daltonics). Cry11Ba GeLC-MS/MS were analyzed with Thermo Proteome Discoverer (VER. 1.4, Thermo Scientific). Native Cry11Aa mass spectrometry data were deconvoluted and analyzed using UniDec. Sequence analysis was carried out using Mascot.

For manuscripts utilizing custom algorithms or software that are central to the research but not yet described in published literature, software must be made available to editors and reviewers. We strongly encourage code deposition in a community repository (e.g. GitHub). See the Nature Portfolio guidelines for submitting code & software for further information.

Data

Policy information about availability of data

All manuscripts must include a data availability statement. This statement should provide the following information, where applicable:

- Accession codes, unique identifiers, or web links for publicly available datasets
- A description of any restrictions on data availability
- For clinical datasets or third party data, please ensure that the statement adheres to our policy

Structures and structure factor amplitudes have been deposited in the PDB databank under accession codes 7QX4 (Cry11Aa WT, pH 7.0; 10.2210/pdb7QX4/pdb), 7QX5 (Cry11Aa Y449F, pH 7.0; 10.2210/pdb7QX5/pdb), 7QX6 (Cry11Aa E583Q, pH 7.0; 10.2210/pdb7QX6/pdb), 7QX7 (Cry11Aa F17Y, pH 7.0; 10.2210/pdb7QX7/pdb), 7QYD (Cry11Ba WT, pH 6.5; 10.2210/pdb7QYD/pdb), 7R1E (Cry11Ba WT, pH 10.4; 10.2210/pdb7R1E/pdb). Raw image files are deposited in cxi.db accession number 190 (https://www.cxidb.org/id-190.html). The source data for Fig. 3 and for Supplementary Figs. 6, 7, 8, 9, 11, 12 and 13 are provided in a combined Source Data file.

Field-specific reporting

Please select the one below that is the best fit for your research. If you are not sure, read the appropriate sections before making your selection.

[x] Life sciences [ ] Behavioural & social sciences [ ] Ecological, evolutionary & environmental sciences

For a reference copy of the document with all sections, see nature.com/documents/nr-reporting-summary-flat.pdf

Life sciences study design

All studies must disclose on these points even when the disclosure is negative.

Sample size	No statistical methods were used to determine sample size. Where applicable, measurements were performed at least in triplicate. Crystallographic data sets have been obtained with high multiplicity. Other sample size were selected based on those accepted in peer-reviewed literature.
Data exclusions	No data were excluded.
Replication	Where applicable, measurements were performed at least in triplicate to ensure reproducibility. The consistency between results obtained in different laboratories with independent sample sources, instruments and data analysis tools supports the reproducibility of the results.
Randomization	Where applicable, measurements were performed randomly.
Blinding	Sample preparation, measurement and analysis was performed by different persons, whenever possible.

Reporting for specific materials, systems and methods

We require information from authors about some types of materials, experimental systems and methods used in many studies. Here, indicate whether each material, system or method listed is relevant to your study. If you are not sure if a list item applies to your research, read the appropriate section before selecting a response.

Materials & experimental systems

n/a	Involved in the study	
[x]	[ ]	Antibodies
[x]	[ ]	Eukaryotic cell lines
[x]	[ ]	Palaeontology and archaeology
[x]	[ ]	Animals and other organisms
[x]	[ ]	Human research participants
[x]	[ ]	Clinical data
[x]	[ ]	Dual use research of concern

Methods

n/a	Involved in the study	
[x]	[ ]	ChIP-seq
[x]	[ ]	Flow cytometry
[x]	[ ]	MRI-based neuroimaging
